# The Copy Number Variation of *OsMTD1* Regulates Rice Plant Architecture

**DOI:** 10.3389/fpls.2020.620282

**Published:** 2021-02-11

**Authors:** Qing Liu, Jinke Xu, Yunhua Zhu, Yuxing Mo, Xue-Feng Yao, Ruozhong Wang, Wenzhen Ku, Zhigang Huang, Shitou Xia, Jianhua Tong, Chao Huang, Yi Su, Wanhuang Lin, Keqin Peng, Chun-Ming Liu, Langtao Xiao

**Affiliations:** ^1^Hunan Provincial Key Laboratory of Phytohormones and Growth Development, College of Bioscience and Biotechnology, Hunan Agricultural University, Changsha, China; ^2^Hengyang Medical College, University of South China, Hengyang, China; ^3^Key Laboratory of Plant Molecular Physiology, Institute of Botany, Chinese Academy of Sciences, Beijing, China; ^4^Institute of Crop Sciences, Chinese Academy of Agricultural Sciences, Beijing, China

**Keywords:** copy number variation, *OsMTD1*, rice, plant architecture, *pri-miR156f*

## Abstract

Copy number variation (CNV) may have phenotypic effects by altering the expression level of the gene(s) or regulatory element(s) contained. It is believed that CNVs play pivotal roles in controlling plant architecture and other traits in plant. However, the effects of CNV contributing to special traits remain largely unknown. Here we report a CNV involved in rice architecture by modulating tiller number and leaf angle. In the genome of *Oryza sativa* ssp. *japonica* cv. Nipponbare, we found a locus *Loc_Os08g34249* is derived from a 13,002-bp tandem duplication in the nearby region of *OsMTD1*, a gene regulating tillering in rice. Further survey of 230 rice cultivars showed that the duplication occurred in only 13 *japonica* rice cultivars. Phenotypic investigation indicated that this CNV region may contribute to tiller number. Moreover, we revealed that *OsMTD1* not only influences rice tiller number and leaf angle, but also represses *pri-miR156f* transcription in the CNV region. Intriguingly, this CNV performs function through both the dosage and position effects on *OsMTD1* and *pri-miR156f*. Thus, our work identified a CNV and revealed a molecular regulatory basis for its effects on plant architecture, implying this CNV may possess importance and application potential in molecular breeding in rice.

## Introduction

Genomic rearrangements include duplications, deletions, and inversions of unique genomic segments at specific regions, as well as translocations, marker chromosomes, isochromosomes, and other complex rearrangements (Lupski, 1998; Feuk et al., 2006; Weckselblatt and Rudd, 2015). These rearrangements are not random events, but instead the reflection of higher-order architectural features of the genome (Lee and Lupski, 2006; Żmien’ko et al., 2014). Different from the whole genome duplication in a cell, the copy number variation (CNV) is the microduplication and deletion, which means an abnormal number of copies of one or more segments of DNA (Sebat et al., 2004). A CNV is commonly regarded as a DNA segment that has been deleted, inserted, or duplicated on certain chromosomes. The length of DNA is more than 1 kb and variable in copy number in comparison with a reference genome (Feuk et al., 2006). Previous studies indicated that CNVs not only involve in intraspecific genome variations, but also cause phenotypic differences. Thus, CNVs can be developed as markers for molecular identification. Genetic diversity can be differentiated by analyzing CNVs (Żmien’ko et al., 2014).

It was reported that CNVs are in variable linkage disequilibrium with flanking SNPs (Hinds et al., 2006; Locke et al., 2006; Yu et al., 2013). CNV could underlie a significant proportion of normal variation including differences in various features (Lee and Lupski, 2006). Known data suggest that CNV mainly affects the members of large families of functionally redundant genes, and the effects of individual CNV events on phenotype are usually modest (Żmien’ko et al., 2014). Altering copy number of a gene family member may only trigger quantitative rather than qualitative changes, making the CNV–phenotype association difficult to be detected. Increasing evidences showed that copy number polymorphisms contribute to natural genetic variation and adaptability in plants; some CNVs for specific genes have been linked to important traits such as flowering time, plant height, and stress resistance (Żmien’ko et al., 2014). A dramatic fruit size change due to a CNV with an insertion of 6–8 kb that affected gene regulation was described during tomato breeding (Cong et al., 2008). In wheat, a CNV has been found to determine the extreme dwarf phenotype by tandem segmental duplication of a region containing the green revolution gene *Rht-D1b* in the haploid genome (Li Y. et al., 2012).

Rice (*Oryza sativa*) is an important staple food crop in the world and a model plant of monocots; whether and how its CNVs are associated with specific traits have also been widely concerned. A CNV at the *GL7* locus has been reported; a tandem duplication of a 17.1-kb segment leads to an increase in grain length (Wang Y. et al., 2015). A 1,212-bp deletion of *qSW5* has been reported to be clearly associated with an increase in rice grain width (Shomura et al., 2008). It has been also reported that a natural tandem array of a 3,137-bp sequence in the upstream of *IPA1* leads to superior yielding (Zhang et al., 2017). Although the knowledge of CNVs in higher plants is still poor, recent studies confirmed the prevalence of CNVs in the *Oryza* species and suggested that CNVs probably play a far more significant role in plant development than previously thought. High-level CNVs existing in different rice cultivars might associate to phenotypic diversity, yet how they affect yield, quality, resistance, and development processes is largely unknown (Li S. et al., 2012; Yu et al., 2013).

*OsMTD1* is a tillering-related gene in rice (Liu et al., 2015). Here we describe a previously unknown transcriptional mechanism that OsMTD1 is able to repress *pri-miR156f* transcripts by the position effect. Furthermore, we provide evidences showing that *OsMTD1*-located region involved a CNV, a tandem segmental duplication resulting in the increasing expression of the *OsMTD1* and reduction of tiller number. This CNV harbors a 13,002-bp region on the eighth chromosome, covering one protein-coding gene *OsMTD1* and a microRNA precursor of osa-miR156f. The results by surveying a panel of 190 rice cultivars showed that 13 of 82 *japonica* cultivars harboring two copies of CNV corresponding sequence by segmental tandem duplication produce less tillers than the one-copy normal cultivars. Transgenic experiments indicated that the *OsMTD1* not only influences tiller number and leaf angle, but also regulates *pri-miR156f* transcription in this CNV region.

## Materials and Methods

### Plant Materials, Field Trails, and Tiller Number Investigation

The mini-core collection accessions from the China National Crop Gene Bank in the Institute of Crop Sciences, Chinese Academy of Agricultural Sciences, as described in Supplementary Table 1, were used in our experiments. Another *japonica* cv. Kitaake was used for CRISPR/Cas9 editing and overexpression analysis. In addition, tobacco (*Nicotiana benthamiana*) leaves were used for *Agrobacterium*-mediated transient expression analysis.

Rice tiller number investigations were conducted in Beijing. Different rice cultivars were transplanted to a paddy field with single plant per hill. The tiller number was counted from three to six randomly chosen individual hills at heading stage in summer of 2011 and autumn of 2013, respectively (Supplementary Table 1).

### Sequence Alignments and Comparisons

The bacterial artificial chromosome sequences from *japonica* cv. Nipponbare and *indica* cv. 93–11 were used to determine the start or the end point range in sequence of *OsMTD1-*located CNV. Then, the 13,002-bp reference genome sequence from Nipponbare was used in BLASTN (National Center for Biotechnology Information) searches against different rice databases for other cultivars, including *japonica* cv. Zhonghua 11 and *indica* cv. Zhenshan 97, Minghui 63, 93–11, Shuhui 498, and RP Bio-226, to determine their orthologous regions. The conserving segments, InDels, and substitution mutations in the orthologous regions of *indica* and *japonica* were identified by using the BLAST, MEGA, and DNAMAN programs.

### Plasmid Construction and Plant Transformation

The vector constructions for the CRISPR/Cas9-mediated gene editing were performed as previously described (Miao et al., 2013). The vectors for *OsMTD1* overexpression in which the *OsMTD1* gene was driven by the CaMV 35S promoter were constructed as previously described (Liu et al., 2015). The constructs were transformed into ZH11 or Kitaake by *Agrobacterium tumefaciens*–mediated transformation (Hiei and Komari, 2008).

Expressions in tobacco leaves were performed in two different plasmids of *pCAMBIA1301* and *pSN1301* vectors using Golden Gate cloning strategy. The *pSN1301* is an adapted form of *pCAMBIA1301* in which a CaMV 35S promoter was added. The region containing the native sequence of *OsMTD1* and *pre-miR156f* was amplified from a *japonica* cv. Nipponbare genomic DNA. The DNA fragment for *pCAM1301::MTD1-OsmiR156f* was amplified by primers 5′-gga tcc ccg ggt acc TGG CAG GTG TAA AGA GGT CA-3′ (prim-177) and 5′-tac gaa ttc gag ctc AAG GAG CAG TTA GAT AAT GGA G-3′ (prim-179) and the DNA fragment for *pSN1301::MTD1-OsmiR156f* was obtained by primers prim-177 and 5′-ggg aaa ttc gag ctc AAG GAG CAG TTA GAT AAT GGA G-3′ (prim-178) and then infused the fragment of interest with *Kpn* I-*Sac* I of *pCAMBIA1301* and *pSN1301* by using ClonExpress II one-step cloning kit (Vazyme, C112-01) to generate plasmid *pCAM1301::MTD1-OsmiR156f* and *pSN1301::MTD1-OsmiR156f*, respectively. The mutant form sequences were obtained by an overlap extension polymerase chain reaction (PCR) method. To generate the *pCAM1301::△MTD1-OsmiR156f* in which *OsMTD1* gene sequence was deleted, primers 5′-gga tcc ccg ggt acc ctt aaa tgc tcc aat agc tag-3′ (prim-182) and prim-179 were used to amplify a fragment sequence from *OsMTD1* gene stop codon to the 60-bp sequence downstream of *pre-miR156f* from genomic DNA, and then the DNA fragment was ligated into the binary vector *pCAMBIA1301* for transformation. Similar strategies were carried out to construct *pCAM1301::ATT-OsmiR156f* in which the ATG start codon of *OsMTD1* was mutated to ATT. The primers 5′-gga tcc ccg ggt acc aga tcg ccg gag atT agc cag aag tc-3′ (prim-183) and prim-179 were used in the ATT mutant fragment amplification. For *pSN1301::△MTD1-OsmiR156f* and the *pSN1301::ATT-OsmiR156f*, a CaMV 35S promoter was harbored at the upstream of *pCAM1301::△MTD1-OsmiR156f* and *pCAM1301::ATT-OsmiR156f*, respectively. The corresponding primers prim-182 and prim-178 were employed for *pSN1301::△MTD1-OsmiR156f*, and prim-183 and prim-178 for *pSN1301::ATT-OsmiR156f*. The constructed vectors were infiltrated into the tobacco leaves by *Agrobacterium tumefaciens*–mediated transformation.

### PCR, Real-Time PCR, and Stem-Loop RT-PCR

Genomic DNA was extracted and purified from fresh young leaves of five plants using CTAB methods. PCR was carried out in a reaction system with a total volume of 20 μL. The primers 5′-ATG AGC CAG AAG TCG TCG TGG C-3′ and 5′-ACA CAT GAA CGT ACA CGG CGC C-3′ were used for *OsMTD1* analysis. PCR validation for CNV was performed in all selected rice cultivars, and three independent experiments were performed for each cultivar. The primers were used as follows: primer64, 5′-AAA TGG CGG AAA CTT GAC AC-3′; primer65, 5′-TGA GCT AGC TGG ACA CAT GG-3′; primer66, 5′-CGG ACC TAA CCA CCG ATC TA-3′; primer67, 5′-ATC TTG GCG CTG CAA TTA TC-3′; inhF, 5′-ATG AGC CAG AAG TCG TCG TGG C-3′; inhR, 5′-ACA CAT GAA CGT ACA CGG CGC C-3′.

Total RNA was isolated from ∼100 mg leaves of five plants using a Trizol reagent (Invitrogen) and treated with RNase-free DNase I (Invitrogen) according to the manufacturer’s instructions. Approximately 5 μg of RNA was used to synthesize first-strand cDNA using poly (dT) oligo primer according to the manufacturer’s instructions in M-MLV kit (Invitrogen). Quantitative real-time reverse transcription PCR (RT-qPCR) was carried out in a reaction system with a total volume of 20 μL, which contained SYBR green I (Invitrogen) on a CFX96 system (BIO-RAD). The following programs were employed: predenaturing for 30 s at 95°C and then amplification for 40 cycles including denaturation for 10 s at 95°C, annealing for 30 s at 60°C, and extension at 72°C for 10 s. The *pri-miR156f* was normalized to the internal rice *tubulinβ-4* gene, and the relative abundance was determined with 2^–Δ^ ^Δ^ ^*Ct*^ method. The RT-qPCR analysis in different lines was repeated three independent times. The primers for testing *pri-miR156f* were 5′-CTT CCC TTC GAC AGG ATA GC-30 and 5′-AGC GGC AGC TGT ATC ATC A-3′.

Stem-loop RT-qPCR (Varkonyi-Gasic et al., 2007) was employed to detect the mature osa-miR156f. Relative expression levels of osa-miR156 were normalized to the internal control *U6* in rice and *NbEF1* in tobacco. PCR was carried out in a reaction system with a total volume of 20 μL, which contained SYBR green I (Invitrogen) on a CFX96 system (BIO-RAD). The following programs were employed: predenaturing for 30 s at 95°C and then amplification for 40 cycles including denaturation for 10 s at 95°C, annealing for 30 s at 60°C, and extension at 72°C for 10 s. The 2^–ΔΔ*Ct*^ method was used to calculate the relative expression level of osa-miR156, and the analysis was repeated three independent times. The primers for *U6* are 5′-TAC AGA TAA GAT TAG CAT GGC CCC-3′ and 5′-GGA CCA TTT CTC GAT TTG TAC GTG-3′, and primers for *NbEF1* are 5′-GAT TGG TGG TAT TGG TAC TGT C-3′ and 5′-AGC TTC GTG GTG CAT CTC-3′.

## Results

### *OsMTD1*-Located Segment Involves a New CNV in Rice

**FIGURE 1:**
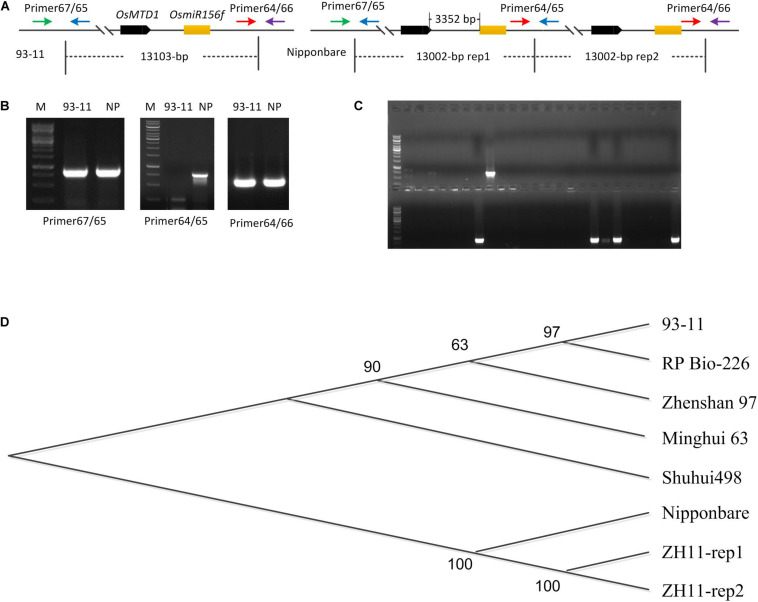
*OsMTD1*-located segment involves a CNV. **(A)** The schematic shows the fragment of *OsMTD1* located on chromosome 8 in 93–11 and Nipponbare. Primer67/65, Primer64/66, and Primer64/65: primer pairs using for tandem duplication event analysis; *OsMTD1* and *OsmiR156f*: the two comprising elements in the corresponding DNA sequence of the *OsMTD1* located CNV. **(B)** Validity analysis on the primers detecting polymorphisms between 93–11 and Nipponbare. **(C)** Partial results from PCR amplification using different rice cultivars. **(D)** Phylogenetic analysis of CNV corresponding region’s sequences in different rice cultivars. Nipponbare: the first sequence of the two DNA segments in *japonica* cv. Nipponbare genome; ZH11-rep1, ZH11-rep2: the first and the second sequence of the two DNA segments in *japonica* cv. ZH11 genome; Shuhui498-rep, 93–11-rep, Minghui 63, RP Bio-226, and Zhanshan97-rep: the corresponding DNA sequence of the *OsMTD1* located-CNV in different *indica* cultivars’ genome.

*OsMTD1* sequence was queried *via* BLAST against four databases: TIGR rice genome annotation^1^, Rice Information GateWay (RIGW^2^), National Center for Biotechnology Information^3^, and the Knowledge-Based Oryza Molecular Biological Encyclopedia (KOME^4^). In the reference genome sequence of *O. sativa* spp. *japonica* cv. *Nipponbare*, we found that another locus, *Loc_Os08g34249*, is identical in DNA sequence to *OsMTD1*, a gene previously reported responsible for tillering in rice. However, it is not the case in the genomes of *indica* cultivars such as 93–11 (Figure 1A) and Shuhui498. Further analysis showed that *OsMTD1* and *Loc_Os08g34249* genes located on rice chromosome 8 according to their positions given in the TIGR rice database. *OsMTD1* and *Loc_Os08g34249* genes coexisted in two overlapped PAC clone AP0082414 and clone AP004703, implying one segmental duplication event on *OsMTD1*. To detect the physical location whereby the CNV event began and ended, we mapped the DNA sequences with different lengths between *OsMTD1* and *Loc_Os08g34249* against the reference genome and found the Nipponbare harbored a 13,002-bp tandem segmental duplication on *OsMTD1*-located region on the eighth chromosome. *OsMTD1* and *Loc_Os08g34249* genes were reciprocal duplication, and each of them was encompassed in a 13,002-bp segment, respectively. Compared with the reference genome of Nipponbare, the tandem duplication in *indica* cultivar 93–11 is absent, and the varied length is more than 1 kb, so the 13,002-bp region encompassing *OsMTD1* could be regarded as a CNV between different rice cultivars. Herein, this DNA segment (about 13,002-bp corresponding region) variation in different rice cultivars was designated as *OsMTD1-*located CNV.

It was reported that the genome sizes of both *indica* and *japonica* subspecies have increased by greater than 2 and 6%, respectively, since their divergence from a common ancestor (Ma and Bennetzen, 2004). To find out whether this CNV contributes to intraspecific genome variations, PCR-amplified corresponding region was employed for a panel of 230 rice cultivars comprising both *indica* and *japonica* subspecies (Supplementary Table 1). The primers were designed according to the genomic sequence of both *japonica* cv. Nipponbare and *indica* cv. 93–11 to distinguish whether a tandem segmental duplication is harbored in the *OsMTD1-*located nearby region. A 754-bp fragment could be amplified from Nipponbare DNA with primer64 and primer65 but not from 93–11 (Figure 1A). The results showed that a clear band was obtained by two primer pairs (primer65 and primer67, primer64, and primer66) in all rice cultivars, representing the flanking sequences of the start or the end points of the corresponding 13,002-bp segment region in Nipponbare, respectively. However, a band was amplified with primer pair of primer64 and primer65 only in the ones whose genome harboring a tandem segmental duplication at the *OsMTD1* gene locus nearby region (Figure 1B). After validation by PCR, only 13 *japonica* cultivars including Nipponbare were found to have a tandem duplication in the corresponding region of *OsMTD1*-located segment (Figure 1C and Supplementary Table 1).

The tandem duplication of *OsMTD1-*located CNV corresponding sequence only appears in some *japonica* cultivars, but not in all investigated *indica* cultivars (Figure 1C and Supplementary Table 1); thus, this CNV represents a large inserted region only in some *japonica* cultivars. We then used the corresponding sequence of *OsMTD1*-located CNV from Nipponbare as a query to search against rice database for other cultivars deposited in National Center for Biotechnology Information (see text footnote 3), including *japonica* cv. Zhonghua11 (ZH11) and *indica* cv. Zhenshan 97, Minghui 63, 93–11, Shuhui 498, and RP Bio-226. Comparative analysis showed that the sequences of *OsMTD1*-located CNV region in different rice cultivars were highly conserved, and the dramatic divergences were found between *japonica* and *indica* subspecies (Figure 1D and Supplementary Table 2). The corresponding fragment of *OsMTD1*-located CNV region includes 107 SNPs, 10 deletions, and 11 insertions, resulting in 111-bp increase in *indica* cv. Shuhui 498, compared with Nipponbare. However, those regions are highly conserved in *indica* cultivars; it reaches 99.96% identity with only a 10-bp deletion and three SNPs among all five *indica* cultivars. As in Nipponbare, a tandem segmental replication at the *OsMTD1*-located regions is found in *japonica* cv. Zhonghua11. However, different from the complete sequence identity of two replication regions in Nipponbare, the sequences of the two DNA segments (designed as rep1 and rep2 according to the order occurred in genome) harbor 35 SNPs or mutations in ZH11, and the identities with the sequence of Nipponbare in rep1 and rep2 are 99.74 and 99.92%, respectively. Distance and Homology matrix analysis using the sequences of *OsMTD1*-located CNV further showed distant evolutionary relationships among different cultivars (Supplementary Table 3).

### Phenotypic Difference According to *OsMTD1*-Located CNV

**FIGURE 2:**
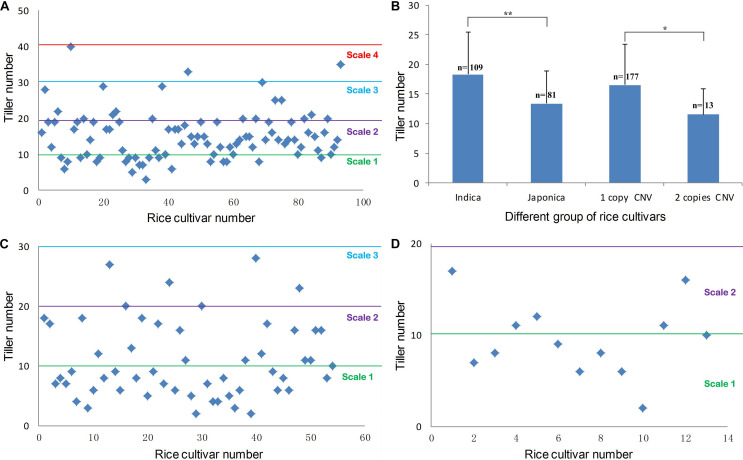
Phenotypic distribution of rice tiller number. **(A)** The maximum tiller number from 94 *indica* accessions. **(B)** The statistical result of the maximum tiller number between *indica* and *japonica* accessions, the maximum tiller number between one-copy and two-copy CNV cultivars. Statistical significance was estimated by Student *t* tests. *P < 0.05 and **P < 0.01. **(C)** The maximum tiller number in different *japonica* accessions with one copy of *OsMTD1* nearby genome sequence. **(D)** The maximum tiller number in different *japonica* accessions with a tandem replication at *OsMTD1* nearby genome sequence.

Copy number variations locating regions that contain protein-coding genes or important regulatory elements often have phenotypic effects (Żmien’ko et al., 2014). Our previous report showed that a T-DNA insertion in *OsMTD1* caused a dramatic change in tiller number (Liu et al., 2015). We therefore postulated this *OsMTD1*-located CNV has effects on a particular architecture trait, i.e., the tiller number. To investigate whether *OsMTD1*-located CNV affects tillering in rice, we performed phenotypic studies using 190 cultivars, including 108 *indica* and 82 *japonica*. Generally, *indica* and *japonica* cultivars show different tillering abilities, so the comparison between the two subspecies provided a reference to judge whether tiller number is a reliable trait for *OsMTD1*-located CNV conveying phenotype analysis. All cultivars were classified into four categories according to the tiller number: scale 1 (<10), scale 2 (10–20), scale 3 (21–30), and scale 4 (>30). Four *indica* while no *japonica* cultivars were classified into scale 4 (Figure 2A). On the contrary, more *japonica* cultivars were classified into scale 1 than *indica* cultivars, amounting to 55.5 and 22.5% of the investigated (Figures 2A,C,D), respectively. The average tiller number in *indica* was apparently higher than that in *japonica* cultivars (Figure 2B), indicating the selected 190 cultivars are a feasible representative group for tillering ability analysis. Further comparative analysis showed that the one-copy normal cultivars produced significantly increased tillers than the tandem duplicated cultivars (Figure 2B). In *japonica* cultivars, 6 of 55 one-copy normal cultivars (10.9%) showed tiller number of scale 3, whereas none was found in 13 two-copy cultivars (Figures 2C,D).

### *OsMTD1*-Located CNV Involves in Rice Plant Architecture

The *OsMTD1*-located CNV region covers about 13,000 bp in different rice cultivars (Supplementary Table 2). GO analysis revealed that, apart from *OsMTD1*, an miR156 family member osa-miR156f was also contained in this CNV, and the *pre-miR156f* sequence located on downstream 3,352-bp away from *OsMTD1* (Figure 1A). The comparative analysis indicated the sequences of *OsMTD1* are identical in all cultivars, and *pre-miR156f* has identical sequences in all *indica* cultivars (Zhenshan 97, Minghui 63, 93–11, Shuhui 498, and RP Bio-226) but shows sequence differences in *japonica* cultivars (Nipponbare and ZH11); however, the final functional sequences of osa-miR156f and osa-miR156f^∗^ are completely identical in all investigated cultivars (Supplementary Figure 1). It is noteworthy that the osa-miR156 was confirmed to be positively correlated with rice tillering (Supplementary Figure 2; Schwab et al., 2005; Xie et al., 2006; Wang L. et al., 2015; Liu et al., 2019), whereas the tiller number comparison in cultivars with different CNV copies showed that the *OsMTD1*-located CNV region negatively affects rice tillering (Figures 2B,D). The osa-miR156f is aggressively antagonistic to *OsMTD1*-located CNV effect on tillering ability, suggesting that *OsMTD1* plays a vital role in the CNV.

**FIGURE 3:**
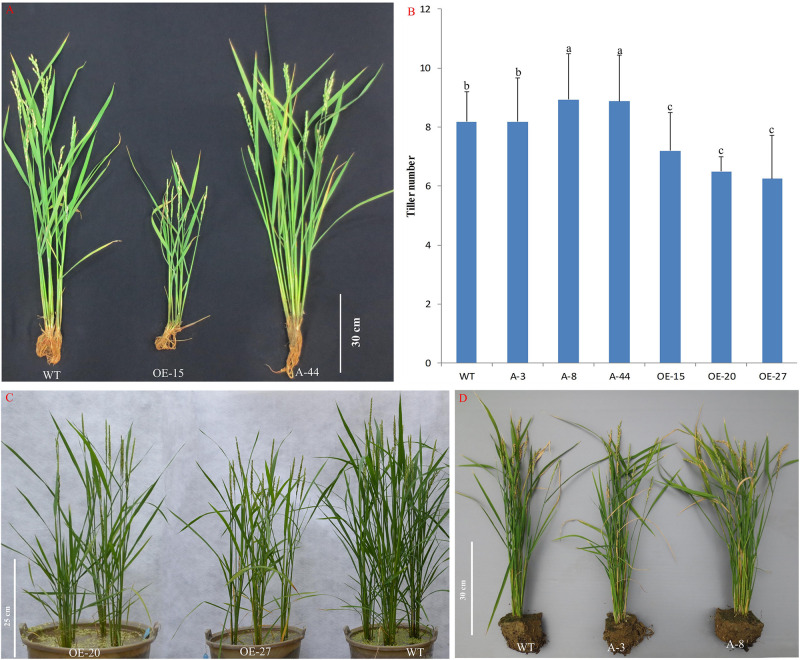
Effects of *OsMTD1* on rice tillering. **(A)** Lines with different *OsMTD1* expression levels and the tillering ability performed. **(B)** Comparative analysis of the tiller number in lines with different *OsMTD1* expression levels. Statistical significance was estimated by Student *t* tests, and different letters indicate a significant difference (*P* < 0.05). **(C)** The *OsMTD1* overexpression lines produce less tillers compared to wild type. **(D)** The *OsMTD1* CRISPR/Cas9 editing lines show different tiller traits. WT: Kitaake; OE-15, OE-20, and OE-27: independent *OsMTD1* overexpression line; A-3, A-8, and A-44: Independent *OsMTD1* CRISPR/Cas9 editing line.

To better understand the role of *OsMTD1* in the CNV, we further analyzed whether *OsMTD1* is directly involved in tiller development; CRISPR/Cas9 genome-editing technology was employed to generate both *Loc_Os08g34249* and *OsMTD1* knockout lines under ZH11 background. In 34 independent T_0_ transgenic lines, sequence analysis revealed that each one belongs to heterogeneity accompanying an A/T/G/C insertion or deletion in *OsMTD1* (Supplementary Figure 3). Surprisingly, no double-knockout mutant was obtained in the CRISPR/Cas9 editing line after self-crossing for four times, and all CRISPR/Cas9 editing lines displayed no obvious phenotypic change. We further carried out CRISPR/Cas9 and overexpression analysis in one copy *japonica* cv. Kitaake and obtained many independent single-base deletion or insertion transgenic lines. All of the single-base mutation lines, in which an A/T/G/C was inserted or deleted, resulting in a frame-shift mutation and the original stop codon of *OsMTD1*, were excluded (Supplementary Table 4). Some mutation lines (such as line A-8 and A-44) significantly increased tiller number, whereas others (such as A-3) showed no difference in tillering ability compared to the wild type. In fact, *OsMTD1* overexpression significantly decreased tiller number (Figure 3), even though the lines showed different *OsMTD1* increased levels (Supplementary Table 5). Intriguingly, the *OsMTD1* overexpression caused multiple phenotypic defects, such as reduction in grain number and plant height, whereas the height of *OsMTD1* CRISPR/Cas9 editing lines was comparable to that of the wild type plants (Supplementary Figure 4). These results under one-copy *OsMTD1*-located CNV background indicated that *OsMTD1* plays a prominent role in the genetic control of tillering ability in rice.

**FIGURE 4:**
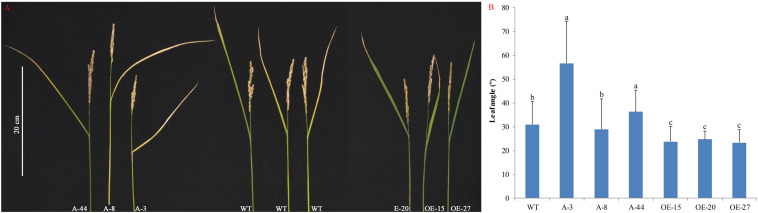
Effects of *OsMTD1* on rice leaf angle. **(A)** Lines with different *OsMTD1* expression levels show different flag leaf angles. **(B)** Comparative analysis the flag leaf angle in lines with different *OsMTD1* expression levels. Statistical significance was estimated by Student *t* tests, and different letters indicate a significant difference (*P* < 0.05). WT: Kitaake; OE-15, OE-20, and OE-27: independent *OsMTD1* overexpression line; A-3, A-8, and A-44: independent *OsMTD1* CRISPR/Cas9 editing line.

Leaf angle is an important agricultural trait determining rice plant architecture and ideotype (Zhou et al., 2017). In our experiments, the results suggest the pivotal role for *OsMTD1* in leaf inclination. Compared with the wild type, some *OsMTD1* CRISPR/Cas9 editing lines (such as A-8) showed no significant impact on leaf inclination at the mature stage, whereas overexpression of *OsMTD1* significantly reduced flag leaf angle (Figure 4). Consistently, the *OsMTD1* RNA*i* lines also presented increasing leaf inclination (Supplementary Figure 2). Conversely, overexpression of *pre-miR156f* increased leaf angle (Supplementary Figure 5). Meanwhile, leaf blades in miR156 knockout lines were found to be more erect than those of the wild type (Miao et al., 2019). These results also indicate that *OsMTD1* and osa-miR156 play opposite roles in regulating leaf angle. Taken together, it could be concluded that *OsMTD1*-located CNV contributes to a compact plant architecture by influencing both tiller number and leaf angle in rice.

### *OsMTD1* Inhibits the Transcript of *MicroRNA156f*

We next explored the underlying molecular mechanism of the *OsMTD1*-located CNV conveying phenotypes. Based on the experimental results mentioned above, an unexpected phenomenon is that the two elements or factors contained in the *OsMTD1*-located CNV region play opposite roles in controlling the architecture *via* tiller number and leaf angle: *OsMTD1* alone negatively regulates while osa-miR156f alone positively modulates these traits.

**FIGURE 5:**
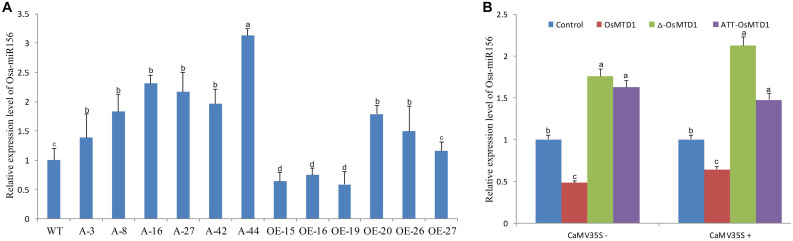
Effects of *OsMTD1* on accumulation of miR156f. **(A)** The osa-miR156 relative levels in different rice lines. WT: Kitaake; A-3, A-8, A-16, A-27, A-42, and A-44: different *OsMTD1* CRISPR/Cas9 editing lines; OE-15, OE-16, OE-19, OE-20, OE-26, OE-27: different *OsMTD1* overexpression lines. **(B)** Quantification of miR156 in tobacco leaves expressing the *pri-miR156f* including *OsMTD1* sequence (OsMTD1), or the *pri-miR156f* in which the *OsMTD1* was deleted (Δ-OsMTD1), or in which the *OsMTD1* start codon was mutated to ATT (ATT-OsMTD1). The empty vector was used as control. Statistical significance was estimated by Student *t* tests, and different letters indicate a significant difference (*P* < 0.05).

The short miRNAs (19–23 nt in length) are processed from corresponding large *pri-miRNAs*. In the large *pri-miRNAs* containing short open reading frame sequences that encode regulatory peptides, this miRNA-encoded peptide (miPEP) increasing the transcription of the *pri-miRNA* was reported (Lauressergues et al., 2015). Because of the close position of the *OsMTD1* to *pri*-*miR156f*, *OsMTD1* might regulate *pri*-*miR156f* transcription. To test this hypothesis, we first analyzed the osa-miR156 levels in both the CRISPR/Cas9 editing and overexpression lines of *OsMTD1* under Kitaake background. The results showed that the CRISPR/Cas9 editing lines produced more osa-miR156 than the wild type (Figure 5). Among the *OsMTD1* overexpression plants, some lines (i.e., OE-15, OE-16, and OE-19) produced less osa-miR156, whereas some lines (i.e., OE-20, OE-26, and OE-27) produced comparable or more osa-miR156 than the wild type.

To further reveal regulatory role of OsMTD1 in osa-miR156f production, we used transformation of tobacco (*Nicotiana tabacum*) leaves to analyze the miR156f level by expressing both the native and mutant promoters of the *pre-miR156f* in *pri-miR156f*. Regardless of CaMV 35S promoter, compared with the amount of miR156f produced by expression of the native *pri-miR156f*, expression of an *OsMTD1* deletion mutant showed higher miR156 abundance. Likewise, expression of a *pri-miR56f* in which the ATG start codon of *OsMTD1* was mutated to ATT also produced higher miR156 level than expression of the native *pri-miR156f* (Figure 5), suggesting that OsMTD1 can inhibit the osa-miR156 accumulation when both are constructed in the same vector.

## Discussion

Copy number variations are major sources of genetic variation influencing gene expression and eventually the phenotype. It is believed that there are more CNVs than chromosome structural variations among individuals, and the total number of nucleotides covered by CNVs is much larger than SNP number in the whole genome (Lupski, 2007; Yu et al., 2013). CNVs can create new genes, change gene dosage, reshape gene structures, and modify elements regulating gene expression (Henrichsen et al., 2009; Zhang et al., 2009). Here, we describe the identification of a new CNV, *OsMTD1*-located CNV, which involves an approximately 13,000-bp tandem duplication in DNA sequence on the eighth chromosome in different rice cultivars (Supplementary Tables 1, 2), and the corresponding sequences of *OsMTD1*-located CNV region in different cultivars are highly conserved, and the sequence includes two important regulator factors, i.e., *OsMTD1* and *pri-miR156f*. The sequence of *OsMTD1*, osa-miR156f, and osa-miR156f^∗^ in all investigated cultivars is identified (Supplementary Figure 1).

Genome-scale studies indicated that CNVs significantly contribute to natural variation in plants (Yu et al., 2013; Żmien’ko et al., 2014; Bai et al., 2016). Changes in gene copy number provide the possibility to rapidly alter the dosage of a gene, which could directly cause a phenotypic variation, and as long as the new beneficial variation being selected over many generations under high selective pressure, the copy number alterations in a particular region may accumulate, and the phenotypic effects may intensify. Segmental duplications longer than 10 kb and of greater than ∼97% sequence identity can lead to local genomic instability (Stankiewicz and Lupski, 2010). As *OsMTD1*-located CNV covering approximately 13,000-bp DNA sequence is an evolutionarily recent duplication in some *japonica* cultivars after highly selective breeding programs, it is not surprising that *OsMTD1*-located CNV contributes to one or more currently advantageous traits in rice. In this article, we have investigated the tiller number on a panel of 190 rice cultivars, and results indicated that this CNV may have phenotypic effects on tiller development; for some, two-copy cultivars produced less tillers than one-copy cultivars (Figure 3). In the study, we also provided evidence that *OsMTD1*-located CNV contains two regulators, i.e., *OsMTD1* and osa-miR156f, jointly regulating tillering and leaf angle (Figures 3, 4; Supplementary Figures 2, 4, 5). Together, these results indicate that *OsMTD1*-located CNV is important for rice plant architecture.

The essential role of a CNV depended on the genes or regulators contained in its region, so the roles of the *OsMTD1*-located CNV in rice phenotypes are determined by its two comprising elements: *OsMTD1* and *pri-miR156f*. In order to reveal the function of *OsMTD1*, the first gene contained in *OsMTD1*-located CNV, CRISPR/Cas9-mediated gene editing technology was employed to knock out *OsMTD1* in the one-copy Kitaake and two-copy ZH11. Some lines both with multiple tillers and large leaf inclination were found under the Kitaake background (Figures 3, 4). Although no obvious tillering phenotype change was observed in the CRISPR/Cas9 editing lines under the two-copy ZH11 background, the reason might be no knockout mutant was obtained in those experiments. Meanwhile, some *OsMTD1* overexpression lines that produced less tiller and smaller leaf angle were obtained (Figures 3, 4). Combining the facts that both the *OsMTD1* RNA*i* lines and mutant lines with a T-DNA insertion into the region of this CNV in two-copy ZH11 displayed multitillering phenotypes (Liu et al., 2015), it is clear that *OsMTD1* alone could be regarded as an executive factor for tillering and leaf angle. For the second gene in *OsMTD1*-located CNV, i.e., *pri-miR156f*, we had demonstrated that the osa-miR156f plays crucial roles in rice tiller development (Liu et al., 2019), which is consistent with previous reports that high-level miR156 causes a bushy phenotype (Schwab et al., 2005; Xie et al., 2006). The effects of *OsmiR156f* on leaf angle are also verified (Supplementary Figure 5; Miao et al., 2019).

How *OsMTD1*-located CNVs regulate rice phenotype is another issue to be explored. It is believed that deletion and duplication can cause a phenotype change *via* several molecular mechanisms, and the commonly recognized mechanism is altering the copy number of a dosage-sensitive gene (or genes) (Lee and Lupski, 2006). The *OsMTD1*-located CNV enclosed two functional elements, *OsMTD1* and *pri-miR156f*; either can act alone as a pleiotropic regulator to determine rice plant architecture in a dosage-dependent manner. However, it seems further explanation is needed for the joint regulation mechanism in plant architecture by *OsMTD1*, *pri-miR156f*, and *OsMTD1*-located CNV. If the CNV phenotype is conveyed by altering *OsMTD1* and *pri-miR156f* dosage only, variation trends of the two contained genes should be the same – both increased or decreased along with the copy number change. In particular, transgenic experiments proved that changing two components of this CNV resulted in contradictory tillering phenotype: compared with the wild type, *OsMTD1* overexpression lines produced less tillers (Figure 3), whereas *pre-miR156f* overexpression lines significantly increased tillers (Supplementary Figure 2). Similarly, *OsMTD1* and *pre-miR156f* overexpressed lines also displayed opposite effects on leaf angle (Figure 4). All experimental data indicated that two genes contained in the CNV region, i.e., *OsMTD1* and *pri-miR156f*, play opposite roles alone in tiller number and leaf angle. Finally, the role of *OsMTD1*-located CNV in rice tillering and leaf angle is apparently consistent with *OsMTD1*, whereas contradictory to osa-miR156f, it was implied that the transcript of *OsMTD1* was more abundant in two-copy cultivars than in one-copy ones, whereas the opposite was true for *pri-miR156f*. Therefore, *OsMTD1* exhibits the major effect and acts as a key factor in the *OsMTD1*-located CNV region and thus contributes to a compact architecture in rice. One possibility is the different extent of genetic buffering, as *pri-miR156f* belongs to a large functionally redundant gene family, and the duplication in the *OsMTD1*-located CNV has only minor effects compared with *OsMTD1*. An alternative explanation is that there might be else unknown factors that inhibit *pri-miR156f* transcription in the CNV.

In addition to changes in gene dosage, many other mechanisms are responsible for the potential effects of CNVs, including reshaping of the gene structure and modification of the elements that regulate gene expression (Henrichsen et al., 2009; Zhang et al., 2009). One possible mechanism is the position effect; i.e., a CNV encompassed regulatory elements might regulate a gene even if they are several Mbs away (Żmien’ko et al., 2014). In the corresponding region of *OsMTD1*-located CNV, *OsMTD1* and *pri-miR156f* are neighboring genes approximately 3.3 kb apart (Figure 1). Hence, we reasoned that *OsMTD1* can inhibit the transcripts of *pri-miR156f via* position effect. Validation for the unpredictable effects of the two distant components in the CNV region is informative. We hypothesized that *OsMTD1* is a regulator repressing *pri-miR156f* transcription and provided some evidence. Compared with wild type, the *OsMTD1* CRISPR/Cas9 editing lines showed higher osa-miR156 level, whereas some *OsMTD1* overexpression lines showed lower osa-miR156 abundance (Figure 5A). Some *OsMTD1* overexpression lines didn’t produce less osa-miR156 than wild type as expected, the reason might be that the insertion location is too far away from *pri-miR156f* in the genome. Furthermore, transformation results in tobacco leaves also showed that the native *pri-miR156f* vector produced less miR156 compared to the deleted and mutated types (Figure 5B). The above evidence implied that OsMTD1 can inhibit its neighboring *pri-miR156f* expression *in vivo* by the position effect. Different from previous report that miRNA-encoded peptide can enhance their corresponding *pri-miRNA* transcription (Lauressergues et al., 2015), OsMTD1 represses *pri-miR156f* transcription. Thus, our work revealed a novel regulatory mechanism for manipulating osa-miR156 level to control tiller number and leaf angle in rice.

## Data Availability Statement

The original contributions presented in the study are included in the article/Supplementary Material, further inquiries can be directed to the corresponding author.

## Author Contributions

QL and LX conceived and designed the research. QL, JX, YZ, YM, and WK performed the experiments. QL, ZH, SX, YS, JT, CH, and WL analyzed the data. JX and X-FY investigated rice tiller number. C-ML provided technical support. QL, RW, KP, and LX wrote the manuscript. All authors read and approved the article.

## Conflict of Interest

The authors declare that the research was conducted in the absence of any commercial or financial relationships that could be construed as a potential conflict of interest.

## Footnotes

1 http://rice.plantbiology.msu.edu/

2 http://rice.hzau.edu.cn/

3 http://www.ncbi.nlm.nih.gov/

4 http://www.cdna01.dna.affrc.go.jp/cDNA

## Supplementary Material

Supplementary Figure 1 The sequence comparison result of *OsMTD1* and pre-*OsmiR156f* in different rice cultivars.

Supplementary Figure 2 The CNV containing components *OsMTD1* and *OsmiR156f* influenced rice tiller development. WT: wild type; OsMTD1-RNAi: *OsMTD1* RNA interference line; OsmiR156f-OE: *OsmiR156f* overexpression line.

Supplementary Figure 3 The *OsMTD1* mutagenesis analysis in rice using CRISPR/Cas9 system under ZH11 background.

Supplementary Figure 4 Effects of *OsMTD1* on rice plant height. Statistical significance was estimated by Student *t* tests, and different letters indicate a significant difference (*P* < 0.05).

Supplementary Figure 5 Effects of *OsmiR156f* on rice leaf angle.

Supplementary Table 1 The surveying of CNV region and tiller number in different rice cultivars.

Supplementary Table 2 DNA polymorphism analysis of *OsMTD1*-located CNV region in various rice cultivars. Nipponbare rep1, Nipponbare rep2: the first and the second sequence of the two DNA segments in Nipponbare genome; ZH11 rep1, ZH11 rep2: the first and the second sequence of the two DNA segments in ZH11 genome; Shuhui 498, 93–11, Minghui 63, RP Bio-226, and Zhenshan 97: the DNA sequence of the *OsMTD1*-located CNV in corresponding *indica* cultivar’s genome.

Supplementary Table 3 The result of Distance and Homology matrix analysis. Evolutionary analysis were conducted in DNAMAN8, and the analysis involved eight sequences: Nipponbare rep1: the first sequence of the two DNA segments in Nipponbare genome; ZH11-rep1, ZH11-rep2: the first and the second sequence of the two DNA segments in ZH11 genome; Shuhui 498, 93–11, Minghui 63, RP Bio-226, and Zhenshan 97: the DNA sequence of the *OsMTD1*-located CNV in corresponding *indica* cultivar’s genome.

Supplementary Table 4 The mutation information of *OsMTD1* in different CRISPR/Cas9 editing lines.

Supplementary Table 5 The *OsMTD1* level in different overexpression lines.

## References

[B1] Bai Z. Chen J. Liao Y. Wang M. Liu R. Ge S. (2016). The impact and origin of copy number variations in the *Oryza* species. *BMC Genom.* 17:261. 10.1186/s12864-016-2589-2 27025496 PMC4812662

[B2] Cong B. Barrero L. S. Tanksley S. D. (2008). Regulatory change in YABBY-like transcription factor led to evolution of extreme fruit size during tomato domestication. *Nat. Genet.* 40 800–804. 10.1038/ng.144 18469814

[B3] Feuk L. Carson A. R. Scherer S. W. (2006). Structural variation in the human genome. *Nat. Rev. Genet.* 7 85–97. 16418744 10.1038/nrg1767

[B4] Henrichsen C. N. Chaignat E. Reymond A. (2009). Copy number variants, diseases and gene expression. *Hum. Mol. Genet.* 18 R1–R8. 19297395 10.1093/hmg/ddp011

[B5] Hiei Y. Komari T. (2008). Agrobacterium-mediated transformation of rice using immature embryos or calli induced from mature seed. *Nat. Protoc.* 3 824–834. 10.1038/nprot.2008.46 18451790

[B6] Hinds D. A. Kloek A. P. Jen M. Chen X. Frazer K. A. (2006). Common deletions and SNPs are in linkage disequilibrium in the human genome. *Nat. Genet.* 38 82–85. 10.1038/ng1695 16327809

[B7] Lauressergues D. Couzigou J.-M. Clemente H. S. Martinez Y. Dunand C. Bécard G. (2015). Primary transcripts of microRNAs encode regulatory peptides. *Nature* 520 90–93. 10.1038/nature14346 25807486

[B8] Lee J. A. Lupski J. R. (2006). Genomic rearrangements and gene copy-number alterations as a cause of nervous system disorders. *Neuron* 52 103–121. 10.1016/j.neuron.2006.09.027 17015230

[B9] Li S. Wang S. Deng Q. Zheng A. Zhu J. Liu H. (2012). Identification of genome-wide variations among three elite restorer lines for hybrid-Rice. *PLoS One* 7:e30952. 10.1371/journal.pone.0030952 22383984 PMC3285608

[B10] Li Y. Xiao J. Wu J. Duan J. Liu Y. Ye X. (2012). A tandem segmental duplication (TSD) in green revolution gene *Rht-D1b* region underlies plant height variation. *New Phytol.* 196 281–291. 10.1111/j.1469-8137.2012.04243.x 22849513

[B11] Liu Q. Shen G. Peng K. Huang Z. Tong J. Kabir M. H. (2015). The alteration in the architecture of a T-DNA insertion rice mutant *osmtd1* is caused by up-regulation of *MicroRNA156f*. *J. Integrat. Plant Biol.* 57 819–829. 10.1111/jipb.12340 25677853 PMC6681133

[B12] Liu Q. Su Y. Zhu Y. Peng K. Hong B. Wang R. (2019). Manipulating *osa-MIR156f* expression by *D18* promoter to regulate plant architecture and yield traits both in seasonal and ratooning rice. *Biol. Proced.* 21:21. 10.1186/s12575-019-0110-4 PMC6827258 31700499

[B13] Locke D. P. Sharp A. J. McCarroll S. A. McGrath S. D. Newman T. L. Cheng Z. (2006). Linkage disequilibrium and heritability of copy-number polymorphisms within duplicated regions of the human genome. *Am. J. Hum. Genet.* 79 275–290. 10.1086/505653 16826518 PMC1559496

[B14] Lupski J. R. (1998). Genomic disorders: structural features of the genome can lead to DNA rearrangements and human disease traits. *Trends Genet.* 14 417–422. 10.1016/s0168-9525(98)01555-8 9820031

[B15] Lupski J. R. (2007). Genomic rearrangements and sporadic disease. *Nat. Genet.* 39 S43–S47. 17597781 10.1038/ng2084

[B16] Ma J. Bennetzen J. L. (2004). Rapid recent growth and divergence of rice nuclear genomes. *Proc. Natl. Acad. Sci. U.S.A.* 101 12404–12410. 10.1073/pnas.0403715101 15240870 PMC515075

[B17] Miao C. Wang Z. Zhang L. Yao J. Hua K. Liu X. (2019). The grain yield modulator miR156 regulates seed dormancy through the gibberellin pathway in rice. *Nat. Commun.* 10:3822. 10.1038/s41467-019-11830-5 PMC6707268 31444356

[B18] Miao J. Guo D. Zhang J. Huang Q. Qin G. Zhang X. (2013). Targeted mutagenesis in rice using CRISPR-Cas system. *Cell Res.* 23 1233–1236. 23999856 10.1038/cr.2013.123 PMC3790239

[B19] Schwab R. Palatnik J. F. Riester M. Schommer C. Schmid M. Weigel D. (2005). Specific effects of microRNAs on the plant transcriptome. *Dev. Cell* 8 517–527. 10.1016/j.devcel.2005.01.018 15809034

[B20] Sebat J. Lakshmi B. Troge J. Alexander J. Young J. Lundin P. (2004). Large-scale copy number polymorphism in the human genome. *Science* 305 525–528. 10.1126/science.1098918 15273396

[B21] Shomura A. Izawa T. Ebana K. Ebitani T. Kanegae H. Konishi S. (2008). Deletion in a gene associated with grain size increased yields during rice domestication. *Nat. Genet.* 40 1023–1028. 10.1038/ng.169 18604208

[B22] Stankiewicz P. Lupski J. R. (2010). Structural variation in the human genome and its role in disease. *Annu. Rev. Med.* 61 437–455. 10.1146/annurev-med-100708-204735 20059347

[B23] Varkonyi-Gasic E. Wu R. Wood M. Walton E. F. Hellens R. P. (2007). Protocol: a highly sensitive RT-PCR method for detection and quantification of microRNAs. *Plant Methods* 3:12. 10.1186/1746-4811-3-12 17931426 PMC2225395

[B24] Wang L. Sun S. Jiye J. Fu D. Yang X. Weng X. (2015). Coordinated regulation of vegetative and reproductive branching in rice. *Proc. Natl. Acad. Sci. U.S.A.* 112 15504–15509. 10.1073/pnas.1521949112 26631749 PMC4687603

[B25] Wang Y. Xiong G. Hu J. Jiang L. Yu H. Xu J. (2015). Copy number variation at the *GL7* locus contributes to grain size diversity in rice. *Nat. Genet.* 47 944–948. 10.1038/ng.3346 26147619

[B26] Weckselblatt B. Rudd M. K. (2015). Human structural variation: mechanisms of chromosome rearrangements. *Trends Genet.* 31 587–599. 10.1016/j.tig.2015.05.010 26209074 PMC4600437

[B27] Xie K. Wu C. Xiong L. (2006). Genomic organization, differential expression, and interaction of SQUAMOSA promoter-binding-like transcription factors and microRNA156 in rice. *Plant Physiol.* 142 280–293. 10.1104/pp.106.084475 16861571 PMC1557610

[B28] Yu P. Wang C. Xu Q. Feng Y. Yuan X. Yu H. (2013). Genome-wide copy number variations in *Oryza sativa* L. *BMC Genom.* 14:649. 10.1186/1471-2164-14-649 24059626 PMC3856455

[B29] Zhang F. Gu W. Hurles M. E. Lupski J. R. (2009). Copy number variation in human health, disease, and evolution. *Annu. Rev. Genom. Hum. Genet.* 10 451–481. 10.1146/annurev.genom.9.081307.164217 PMC4472309 19715442

[B30] Zhang L. Yu H. Ma B. Liu G. Wang J. Wang J. (2017). A natural tandem array alleviates epigenetic repression of *IPA1* and leads to superior yielding rice. *Nat. Commun.* 8:14789. 10.1038/ncomms14789 PMC5364388 28317902

[B31] Zhou L. Xiao L. Xue H. (2017). Dynamic cytology and transcriptional regulation of rice lamina joint development. *Plant Physiol.* 174 1728–1746. 10.1104/pp.17.00413 28500269 PMC5490912

[B32] Żmien’ko A. Samelak A. Kozłowski P. Figlerowicz M. (2014). Copy number polymorphism in plant genomes. *Theor. Appl. Genet.* 127 1–18. 10.1007/s00122-013-2177-7 23989647 PMC4544587

