# Supplementary figures and images for: The Copy Number Variation of OsMTD1 Regulates Rice Plant Architecture
Source: Front Plant Sci. 2021 Feb 11;11:620282. doi: 10.3389/fpls.2020.620282 (PMC7905320; doi:10.3389/fpls.2020.620282)

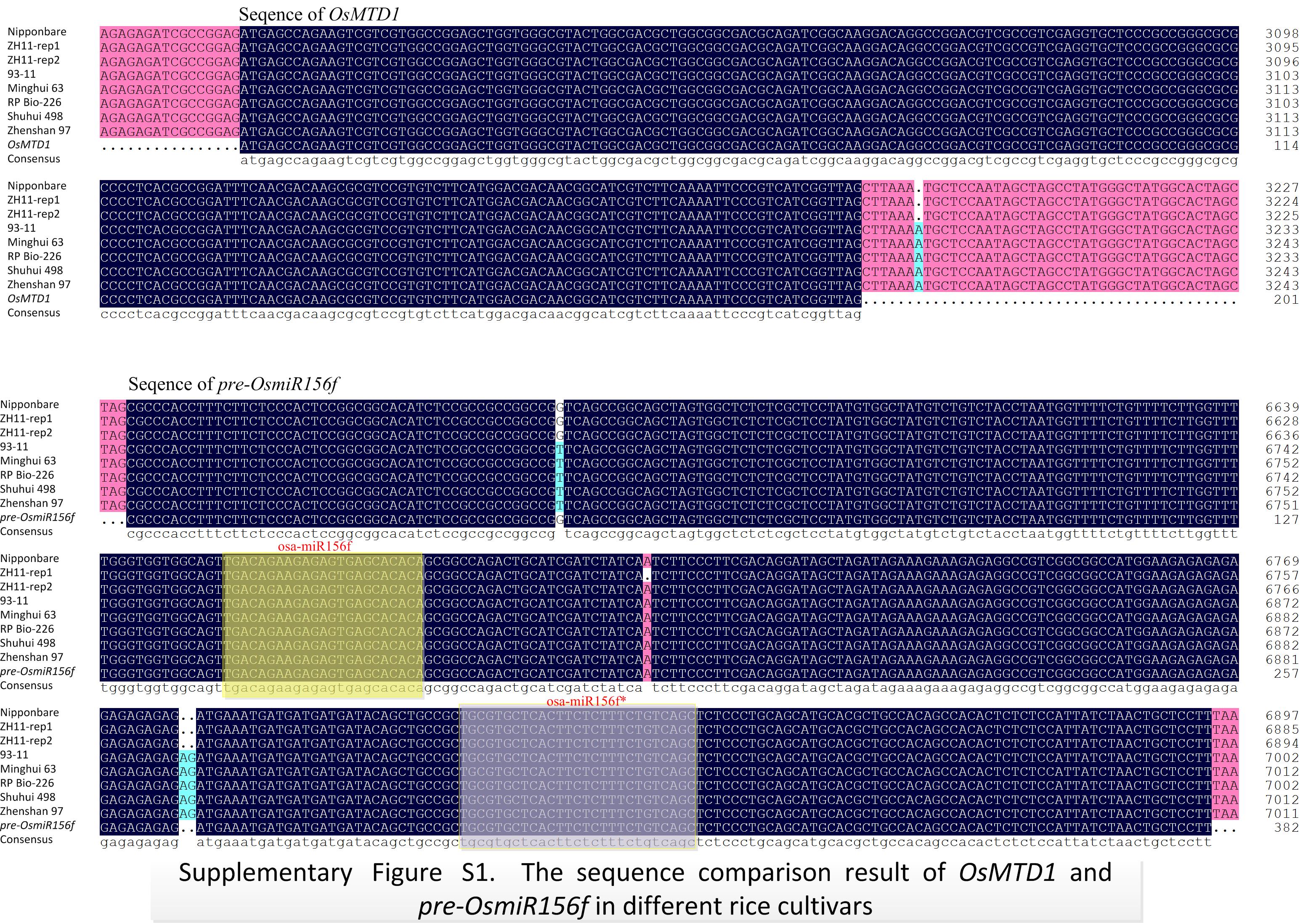

Supplement: Supplementary Figure 1 — The sequence comparison result of OsMTD1 and pre-OsmiR156f in different rice cultivars. [file Image_1.JPEG]

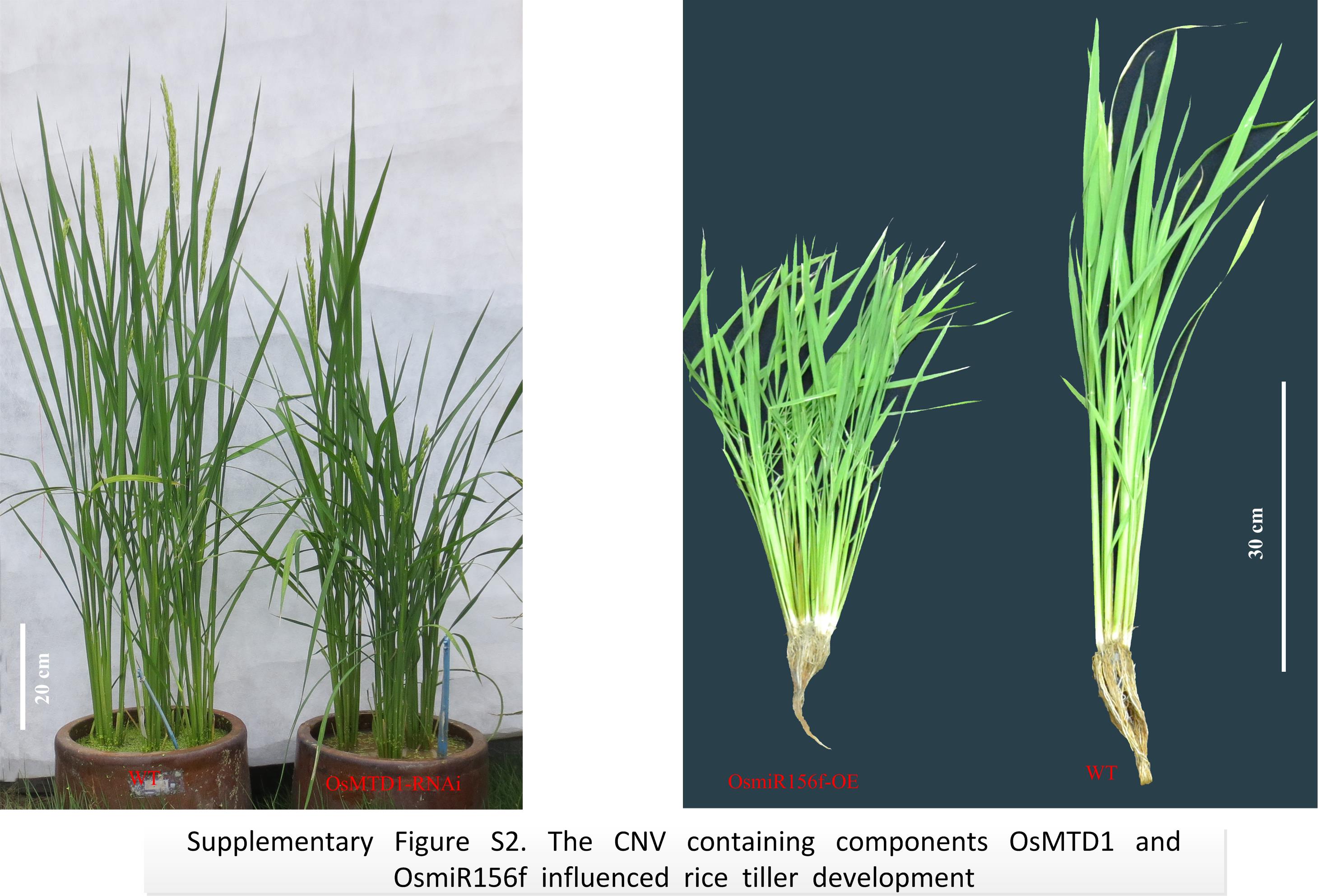

Supplement: Supplementary Figure 2 — The CNV containing components OsMTD1 and OsmiR156f influenced rice tiller development. WT: wild type; OsMTD1-RNAi: OsMTD1 RNA interference line; OsmiR156f-OE: OsmiR156f overexpression line. [file Image_2.JPEG]

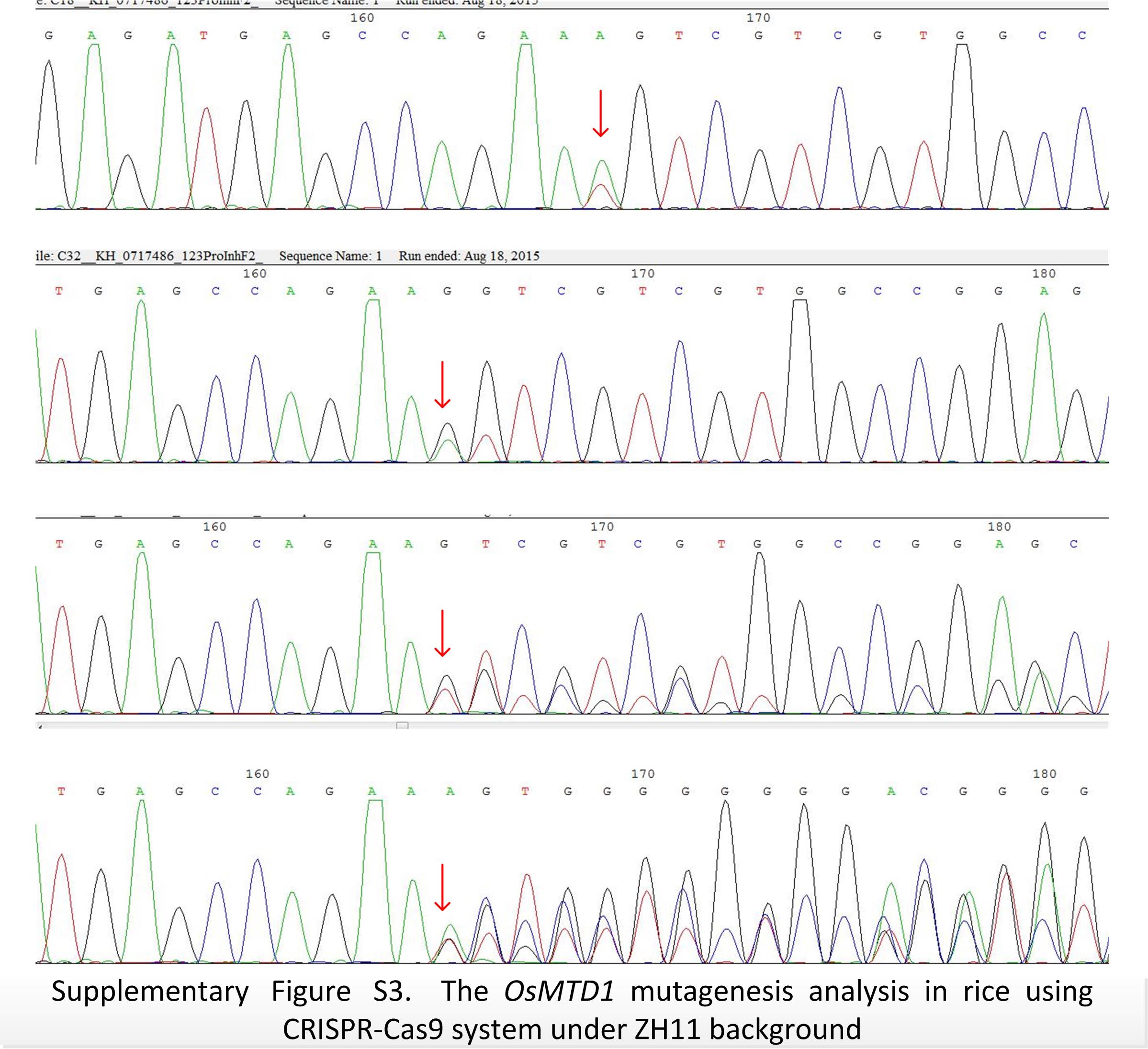

Supplement: Supplementary Figure 3 — The OsMTD1 mutagenesis analysis in rice using CRISPR/Cas9 system under ZH11 background. [file Image_3.JPEG]

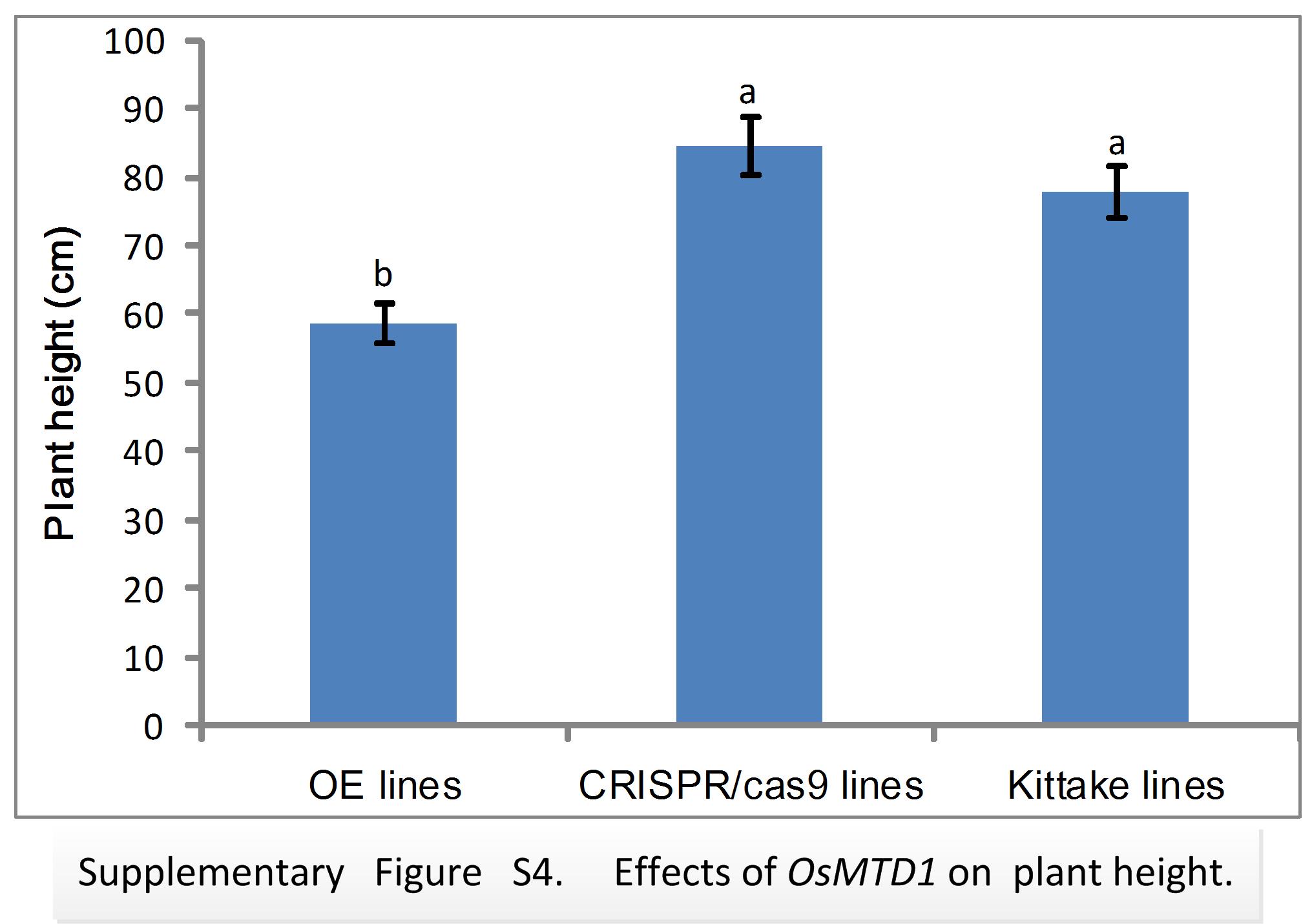

Supplement: Supplementary Figure 4 — Effects of OsMTD1 on rice plant height. Statistical significance was estimated by Student t tests, and different letters indicate a significant difference (P < 0.05). [file Image_4.JPEG]

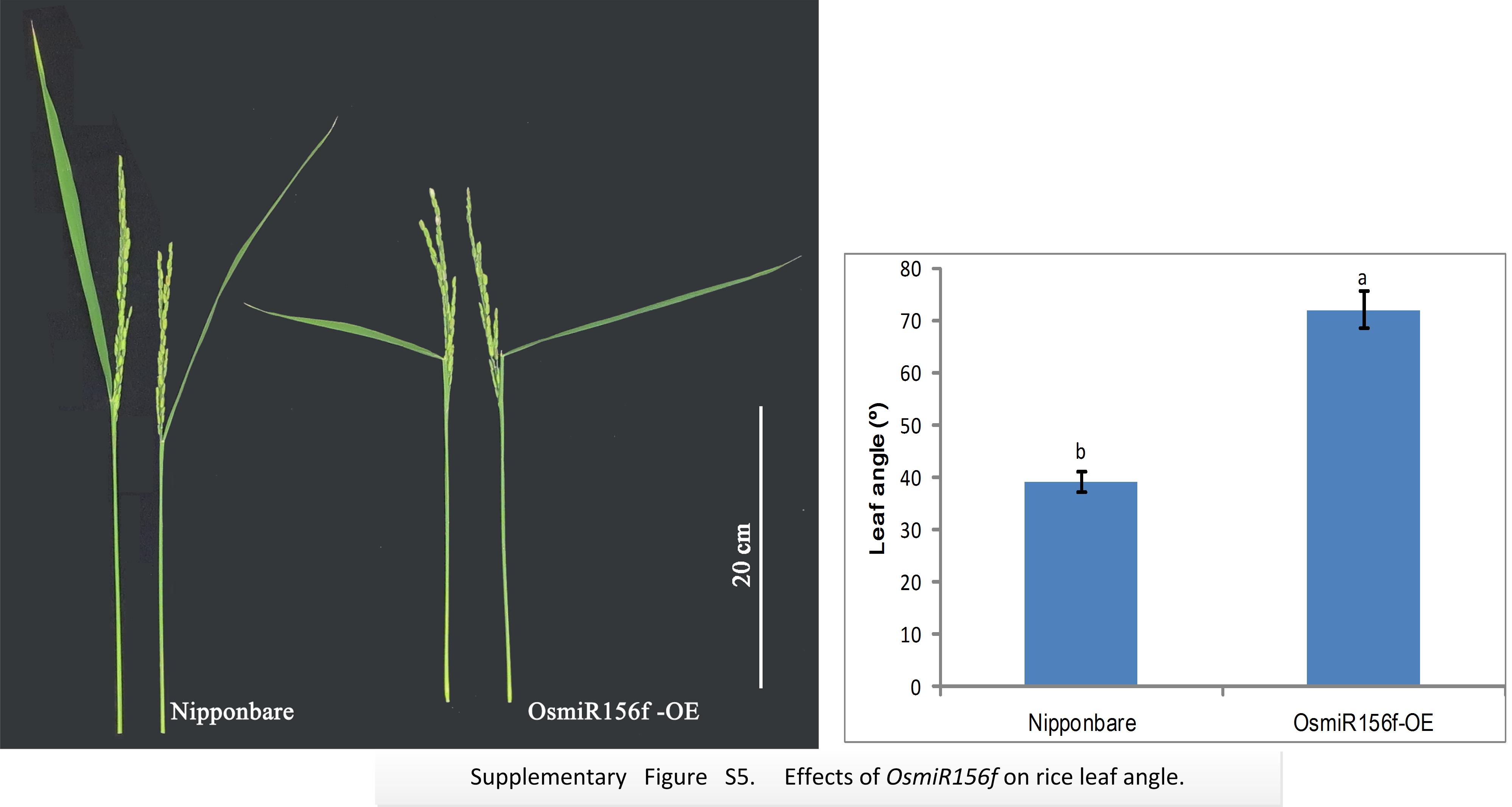

Supplement: Supplementary Figure 5 — Effects of OsmiR156f on rice leaf angle. [file Image_5.JPEG]
