# Supplementary material for: The Copy Number Variation of OsMTD1 Regulates Rice Plant Architecture
Source: Front Plant Sci. 2021 Feb 11;11:620282. doi: 10.3389/fpls.2020.620282 (PMC7905320; doi:10.3389/fpls.2020.620282)
Supplement: Supplementary Table 1 — The surveying of CNV region and tiller number in different rice cultivars. [file Table_1.DOCX]

**Supplement Table 1** The surveying of CNV region and tiller number in different rice cultivars

| Code number |  | Accession  number | Chinese name of variety | Variety name | Subspecies  kind | CNV region  number | Tiller number |
| --- | --- | --- | --- | --- | --- | --- | --- |
| 1 |  | 02-00058 | 抚宁紫皮粳子 | Funingzipigengzi | Japonica | 1 | -/18 ^*^ |
| 2 |  | 02-00133 | 隆化葫芦 | Longhuahulu | Japonica | 1 | 18/15 |
| 3 |  | 02-00210 | 高阳淀稻 | Gaoyangdiandao | Japonica | 2 | 17/18 |
| 4 |  | 02-00294 | 水原300粒 | Shuiyuan 300Li | Japonica | 2 | 7/12 |
| 5 |  | 02-00295 | 叶里藏花 | Yelicanghua | Japonica | 2 | 8/15 |
| 7 |  | 05-00024 | 卫国 | Weiguo | Japonica | 1 | -/16 |
| 8 |  | 05-00052 | 丹东陆稻 | Dandongludao | Japonica | 1 | 7/11 |
| 9 |  | 06-00035 | 兴国 | Xingguo | Japonica | 1 | 9/13 |
| 10 |  | 07-00010 | 老光头83 | Laoguangtou 83 | Japonica | 1 | -/17 |
| 12 |  | 08-00036 | 木樨球 | Muxiqiu | Japonica | 1 | 4/14 |
| 13 |  | 08-00066 | 老虎种 | Laohuzhong | Japonica | 1 | 18/15 |
| 15 |  | 09-00530 | 黄壳早二十 | Huangkezaoershi | Japonica | 1 | 3/10 |
| 16 |  | 09-00724 | 百歌稻 | Baigedao | Japonica | 1 | 6/16 |
| 17 |  | 09-01361 | 寸三粒 | Cunsanli | Japonica | 1 | -/11 |
| 19 |  | 11-00322 | 六十早 | Liushizao | Indica | 1 | 16/22 |
| 20 |  | 11-00389 | 秋千白 | Qiuqianbai | Indica | 1 | 28/14 |
| 21 |  | 11-00403 | 雷火占 | Leihuozhan | Indica | 1 | 19/22 |
| 22 |  | 11-00529 | 肥东塘稻 | Feidongtangdao | Japonica | 1 | 12/- |
| 23 |  | 12-00589 | 金溪白 | Jinxibai | Indica | 1 | 12/21 |
| 24 |  | 12-00644 | 解放籼 | Jiefangxian | Indica | 1 | 19/19 |
| 25 |  | 12-01446 | 三百粒 | Sanbaili | Indica | 1 | -/18 |
| 26 |  | 12-02254 | 台山糯 | Taishannuo | Indica | 1 | -/9 |
| 27 |  | 12-02280 | 矮禾迟 | Aihechi | Indica | 1 | 22/24 |
| 28 |  | 12-02373 | 矮密 | Aimi | Indica | 1 | 9/10 |
| 29 |  | 12-02850 | 红米三担 | Hongmisandan | Japonica | 1 | 8/15 |
| 30 |  | 13-00723 | 金包银 | Jinbaoyin | Japonica | 1 | 27/20 |
| 31 |  | 13-00737 | 闽北晚籼 | Minbeiwanxian | Indica | 1 | 6/- |
| 32 |  | 13-00816 | 陆财号 | Lucaihao | Indica | 1 | 8/10 |
| 33 |  | 13-01006 | 乌壳占 | Wukezhan | Indica | 1 | 40/17 |
| 35 |  | 13-01433 | 盐水赤 | Yanshuichi | Indica | 1 | -/14 |
| 36 |  | 15-00503 | 鼠牙占 | Shuyazhan | Indica | 1 | 17/17 |
| 37 |  | 15-00648 | 丝苗 | Simiao | Indica | 1 | 19/- |
| 38 |  | 15-01740 | 饿死牛 | Esiniu | Indica | 1 | 9/22 |
| 39 |  | 15-03025 | 齐眉 | Qimei | Indica | 1 | 20/15 |
| 40 |  | 15-03057 | 南雄早油占 | Nanxiongzaoyouzhan | Indica | 1 | 10/- |
| 41 |  | 15-03168 | 白壳花螺 | Baikehualuo | Indica | 1 | 14/16 |
| 42 |  | 15-03336 | 黑督4 | Heidu 4 | Indica | 1 | 19/- |
| 43 |  | 15-03586 | 赤壳糯 | Chikenuo | Indica | 1 | 8/18 |
| 44 |  | 15-04016 | 三粒寸 | Sanlicun | Japonica | 1 | 9/10 |
| 45 |  | 15-04286 | 西什15 | Xishi 15 | Japonica | 1 | 6/- |
| 46 |  | 16-00163 | 横县良春 | Hengxianliangchun | Indica | 1 | 9/- |
| 47 |  | 16-01841 | 矮仔占 | Aizizhan | Indica | 1 | 29/21 |
| 48 |  | 16-02459 | 红粳旱谷 | Honggenghangu | Japonica | 1 | 20/- |
| 49 |  | 16-05252 | 红矮糯 | Hongainuo | Indica | 1 | 17/- |
| 50 |  | 16-06887 | 七月籼 | Qiyuexian | Indica | 1 | 17/- |
| 51 |  | 16-09350 | 光壳香糯 | Guangkexiangnuo | Japonica | 1 | -/14 |
| 53 |  | 17-00502 | 洞庭晚籼 | Dongtingwanxian | Indica | 1 | 21/- |
| 54 |  | 17-00524 | 柳叶粘 | Liuyenian | Indica | 1 | 22/14 |
| 55 |  | 17-00966 | 宜恩长坛 | Yienchangtan | Indica | 1 | 19/18 |
| 56 |  | 17-01470 | 霸王鞭1 | Bawangbian 1 | Japonica | 1 | 13/- |
| 58 |  | 18-01903 | 须谷糯 | Xugunuo | Japonica | 1 | 8/- |
| 59 |  | 18-03950 | 木瓜糯 | Muguanuo | Japonica | 1 | -/13 |
| 60 |  | 18-04082 | 红旗5号 | Hongqi 5 | Japonica | 1 | -/8 |
| 61 |  | 18-04906 | 万利籼 | Wanlixian | Indica | 1 | 11/19 |
| 62 |  | 19-00022 | 香稻 | Xiangdao | Indica | 1 | -/15 |
| 63 |  | 19-00205 | 旱麻稻 | Hanmadao | Indica | 1 | -/10 |
| 64 |  | 20-01262 | 细白粘 | Xibaizhan | Japonica | 1 | 18/- |
| 65 |  | 20-01452 | 麻麻谷 | Mamagu | Indica | 1 | 8/14 |
| 67 |  | 20-02073 | 梅花糯 | Meihuanuo | Indica | 1 | 9/21 |
| 69 |  | 20-03042 | 红谷 | Honggu | Indica | 1 | 5/18 |
| 70 |  | 20-03053 | 三颗寸 | Sankecun | Indica | 1 | 9/- |
| 71 |  | 20-03215 | 山酒谷 | Shanjiugu | Japonica | 1 | 5/- |
| 72 |  | 21-00083 | 毫马克（K） | Haomake (K) | Japonica | 1 | 9/17 |
| 73 |  | 21-00272 | 文香糯 | Wenxiangnuo | Indica | 1 | 7/- |
| 74 |  | 21-00357 | 毫补卡 | Haobuka | Japonica | 1 | 17/19 |
| 76 |  | 21-00785 | 本邦谷 | Benbanggu | Japonica | 1 | 7/12 |
| 77 |  | 21-01082 | 大弯糯 | Dawannuo | Indica | 1 | -/17 |
| 78 |  | 21-01106 | 紫米 | Zimi | Japonica | 1 | -/7 |
| 79 |  | 21-01120 | 香谷 | Xianggu | Japonica | 1 | -/14 |
| 80 |  | 21-01165 | 小红谷 | Xiaohonggu | Japonica | 1 | 24/17 |
| 81 |  | 21-01257 | 清可 | Qingke | Indica | 1 | 7/15 |
| 82 |  | 21-01577 | 五子堆 | Wuzidui | Japonica | 1 | -/9 |
| 83 |  | 21-01744 | 老造谷 | Laozaogu | Indica | 1 | 3/15 |
| 84 |  | 21-01853 | 毫巴永1 | Haobayong 1 | Japonica | 1 | 6/- |
| 85 |  | 21-01899 | 公居73 | Gongju 73 | Japonica | 1 | 16/10 |
| 86 |  | 21-01970 | 冷水谷2 | Lengshuigu 2 | Japonica | 1 | -/5 |
| 87 |  | 21-01989 | 冷水糯 | Lengshuinuo | Japonica | 1 | -/11 |
| 89 |  | 21-02171 | 齐头谷 | Qitougu | Indica | 1 | 9/- |
| 90 |  | 21-02224 | 紫糯 | Zinuo | Indica | 1 | -/21 |
| 93 |  | 21-02769 | 魔王谷内 | Mowanggunei | Japonica | 1 | 11/- |
| 94 |  | 21-02824 | 毫菜 | Haocai | Indica | 1 | 20/9 |
| 97 |  | 21-03121 | 南高谷 | Nangaogu | Indica | 1 | 11/- |
| 98 |  | 21-03433 | 金枝糯 | Jinzhinuo | Indica | 1 | 9/- |
| 99 |  | 21-03781 | 鸡血糯 | Jixuenuo | Japonica | 1 | 5/- |
| 100 |  | 21-03879 | 饭毫皮 | Fanhaopi | Indica | 1 | 29/- |
| 102 |  | 21-04506 | 枇五升 | Piwusheng | Indica | 1 | 10/15 |
| 105 |  | 21-05072 | 乌珇红谷 | Wuzuhonggu | Indica | 1 | 17/- |
| 106 |  | 21-05171 | 背子糯 | Beizinuo | Japonica | 1 | 20/- |
| 107 |  | 22-00040 | 泽谷 | Zegu | Japonica | 1 | 7/11 |
| 109 |  | 22-00570 | 香糯 | Xiangnuo | Japonica | 1 | 4/16 |
| 110 |  | 22-01439 | 粘壳糯 | Zhankenuo | Indica | 1 | 6/- |
| 111 |  | 22-01615 | 马尾粘 | Maweizhan | Indica | 1 | -/15 |
| 113 |  | 22-02148 | 寸谷糯 | Cungunuo | Indica | 1 | -/13 |
| 115 |  | 22-02423 | 紫芒飞蛾 | Zimangfeie | Japonica | 1 | 4/- |
| 116 |  | 22-02754 | 油粘 | Youzhan | Japonica | 1 | 8/14 |
| 117 |  | 22-03815 | 贯推白禾1 | Guantuibaihe 1 | Japonica | 1 | 5/- |
| 118 |  | 22-04053 | 阳壳糯 | Yangkenuo | Indica | 1 | -/17 |
| 119 |  | 22-04574 | 毫虑光粘 | Haolvguangzhan | Japonica | 1 | -/12 |
| 120 |  | 22-04637 | 小白米 | Xiaobaimi | Indica | 1 | -/15 |
| 122 |  | 24-00215 | 老红稻 | Laohongdao | Japonica | 1 | 6/7 |
| 123 |  | 26-00008 | 加巴拉 | Jiabala | Indica | 1 | 17/16 |
| 124 |  | 28-00005 | 黑芒稻 | Heimangdao | Japonica | 1 | -/13 |
| 125 |  | 29-00010 | 葡萄黄 | Putaohuang | Japonica | 2 | 11/16 |
| 126 |  | 30-00195 | 台东陆稻 | Taidongludao | Japonica | 1 | -/13 |
| 127 |  | 30-00206 | 台中65号 | Taizhong 65 | Japonica | 2 | 2/- |
| 128 |  | 30-00210 | 台中在来1 | Taizhongzailai 1 | Indica | 1 | 17/28 |
| 129 |  | 30-00244 | 台中籼选2 | Taizhongxianxuan 2 | Indica | 1 | 13/- |
| 130 |  | 31-00032 | 闷加高1 | Menjiagao 1 | Japonica | 1 | 28/- |
| 131 |  | 31-00042 | 闷加丁2 | Menjiading 2 | Indica | 1 | 18/19 |
| 132 |  | 31-00388 | 包二幅 | Baoerfu | Indica | 1 | 33/- |
| 133 |  | A0016 | 金南特B | Jinnante B | Indica | 1 | 15/40 |
| 134 |  | A0060 | 竹真B | Zhuzhen B | Indica | 1 | 13/- |
| 135 |  | A0086 | 朝阳一号B | Chaoyangyihao B | Indica | 1 | 15/35 |
| 136 |  | A0096 | L 301B | L 301B | Indica | 1 | 19/34 |
| 137 |  | A00112 | 安农晚粳B | Annongwangeng B | Japonica | 1 | -/17 |
| 138 |  | A00120 | 金南特43B | Jinnante 43B | Indica | 1 | 15/21 |
| 139 |  | A00132 | 早熟农虎6 | Zaoshunonghu 6 | Japonica | 2 | 12/- |
| 140 |  | A00172 | 青四矮16B | Qingsiai 16B | Indica | 1 | 13/23 |
| 141 |  | A00240 | 珍汕97B | Zhenshan 97B | Indica | 1 | 8/15 |
| 142 |  | A00244 | 献改B | Xiangai B | Indica | 1 | 10/9 |
| 143 |  | A00246 | 江农早1号 | Jiangnongzao 1 B | Indica | 1 | 19/13 |
| 144 |  | A00298 | 京虎B | Jinghu B | Japonica | 1 | -/28 |
| 145 |  | A00386 | 黎明B | Liming B | Japonica | 1 | -/15 |
| 146 |  | A00408 | 滇瑞409B | Dianrui 409B | Indica | 1 | 12/- |
| 147 |  | A00430 | 包协123B | Baoxie 123B | Indica | 1 | 8/14 |
| 148 |  | A00434 | 80B | 80B | Indica | 1 | 8/- |
| 149 |  | A00464 | 包协-7B | Baoxie -7B | Indica | 1 | -/20 |
| 150 |  | A00596 | G珍汕97B | G Zhenshan 97B | Japonica | 1 | 17/- |
| 152 |  | R0004 | 古154 | Gu 154 | Indica | 1 | 12/24 |
| 153 |  | R00014 | 圭630 | Gui 630 | Indica | 1 | -/22 |
| 154 |  | R00032 | IR661-1 | IR661-1 | Indica | 1 | 10/14 |
| 155 |  | R00015 | 培C122 | Pei C122 | Japonica | 2 | 9/- |
| 156 |  | R0333 | 粳7623 | Geng 7623 | Japonica | 2 | 6/11 |
| 157 |  | R0337 | 宁恢21 | Ninghui 21 | Japonica | 2 | 8/- |
| 158 |  | R0430 | 76-1 | 76-1 | Japonica | 2 | 6/- |
| 160 |  | R0468 | 特青选恢 | Teqingxuanhui | Indica | 1 | 13/19 |
| 161 |  | R0515 | 湘恢91269 | Xianghui 91269 | Indica | 1 | 14/15 |
| 163 |  | ZD-00002 | 广陆矮4号 | Guanglu Ai 4 | Indica | 1 | 20/- |
| 164 |  | ZD-00141 | 矮脚南特 | Aijiaonante | Indica | 1 | 15/24 |
| 165 |  | ZD-00213 | 柳沙1号 | Liusha 1 | Indica | 1 | 15/17 |
| 166 |  | ZD-00358 | 郴晚3号 | Chenwan 3 | Japonica | 1 | 16/19 |
| 167 |  | ZD-00474 | 二九南1号 | Erjiunan 1 | Indica | 1 | 12/19 |
| 168 |  | ZD-00560 | 南京11号 | Nanjing 11 | Indica | 1 | 20/17 |
| 171 |  | ZD-00743 | 成都矮3号 | Chengduai 3 | Indica | 1 | 8/16 |
| 172 |  | ZD-00747 | 矮麻抗 | Aimakang | Indica | 1 | 30/12 |
| 173 |  | ZD-00760 | 蜀丰101 | Shufeng 101 | Indica | 1 | 14/19 |
| 174 |  | ZD-00806 | 立新粳 | Lixingeng | Japonica | 1 | 23/- |
| 175 |  | ZD-01001 | 黑粳2号 | Heigeng 2 | Japonica | 1 | -/10 |
| 176 |  | ZD-01006 | 桂朝2号 | Guizhao 2 | Indica | 1 | 19/19 |
| 177 |  | ZD-01108 | 二钢矮 | Ergangai | Indica | 1 | 16/15 |
| 178 |  | ZD-01195 | 包选21号 | Baoxuan 21 | Indica | 1 | 25/14 |
| 179 |  | ZD-01266 | 广陆矮15- | Guangluai 15- | Indica | 1 | 14/12 |
| 180 |  | ZD-01328 | 红晚1号 | Hongwan 1 | Indica | 1 | 25/- |
| 181 |  | ZD-01402 | 湘矮早10 | Xiangaizao 10 | Indica | 1 | 13/23 |
| 182 |  | ZD-01423 | 湘晚籼1号 | Xiangwanxian 1 | Indica | 1 | 14/- |
| 185 |  | ZD-01820 | 扬稻2号 | Yangdao 2 | Indica | 1 | 19/- |
| 186 |  | ZD-02017 | 泸科3号 | Luke 3 | Indica | 1 | 14/12 |
| 187 |  | ZD-02032 | 矮沱谷151 | Aituogu 151 | Indica | 1 | 10/19 |
| 188 |  | ZD-02261 | 中花8号 | Zhonghua 8 | Japonica | 1 | -/15 |
| 189 |  | ZD-02277 | 晋稻1号 | Jindao 1 | Japonica | 1 | -/12 |
| 190 |  | ZD-023241 | 辽粳287 | Liaogeng 287 | Japonica | 1 | -/20 |
| 191 |  | ZD-02431 | 黄丝桂占 | Huangsiguizhan | Indica | 1 | 12/- |
| 193 |  | ZD-02547 | 墨米 | Momi | Indica | 1 | 20/21 |
| 194 |  | ZD-02605 | 金优1号 | Jinyou 1 | Indica | 1 | 16/30 |
| 195 |  | ZD-02685 | 粳87-304 | Geng 87-304 | Japonica | 1 | -/16 |
| 196 |  | ZD-02694 | 湘晚籼3号 | Xiangwanxian 3 | Indica | 1 | 21/- |
| 197 |  | ZD-02715 | 湘早籼7号 | Xiangzaoxian 7 | Indica | 1 | 15/10 |
| 198 |  | ZD-02944 | 镇籼232 | Zhenxian 232 | Indica | 1 | 11/22 |
| 199 |  | ZD-03104 | 早籼240 | Zaoxian 240 | Indica | 1 | 9/- |
| 200 |  | ZD-03115 | 当育5号 | Dangyu 5 | Japonica | 1 | 11/10 |
| 201 |  | ZD-03386 | 成农水晶 | Chengnongshuijing | Indica | 1 | 16/6 |
| 202 |  | ZD-03525 | 郑稻5号 | Zhengdao 5 | Japonica | 1 | -/14 |
| 204 |  | WT-1 | 中花11 | ZH11 | Japonica | 2 | 11/15 |
| 205 |  | WT-2 | 秀水63 | Xiushui63 | Japonica | 1 | 16/13 |
| 207 |  | WT-4 | 南京6号 | NanJing 6 | Indica | 1 | 20/33 |
| 208 |  | WT-5 | 日本晴 | Nipponbare | Japonica | 2 | 16/13 |
| 209 |  | WT-6 | 特青 | Teqing | Indica | 1 | -/16 |
| 210 |  | WT-7 | 旱稻65 | Handao 65 | Japonica | 1 | 8/7 |
| 211 |  | WT-8 | ZF-802 | ZF-802 | Indica | 1 | 10/24 |
| 212 |  | WT-9 | 95-22 | 95-22 | Japonica | 1 | -/16 |
| 213 |  | WT-10 | DonJin | DonJin | Japonica | 2 | 10/6 |
| 214 |  | WT-11 | 93-11 | 93-11 | Indica | 1 | 12/- |
| 215 |  | WT-12 | 龙特普 | Longtepu | Indica | 1 | 14/10 |
| 216 |  | WT-13 | Kasalath | Kasalath | Indica | 1 | 35/22 |
| 217 |  | WT-14 | Bala | Bala | Indica | 1 | -/13 |
| 218 |  | WT-15 | Pokkali | Pokkali | Japonica | 1 | -/6 |
| 221 |  | WT-18 | Cabacu | Cabacu | Japonica | 1 | -/16 |
| 222 |  | WT-19 | IRAT109 | IRAT109 | Japonica | 1 | -/15 |
| 223 |  | WT-20 | T309 | T309 | Japonica | 1 | -/20 |
|  |  |  |  |  |  |  |  |
| 6 |  | 04-00115 | 叶楼一号1 | Yelou 1 | Japonica | 1 | -/- |
| 11 |  | 07-00109 | 白毛稻 | Baimaodao | Japonica | 1 | -/- |
| 14 |  | 08-00253 | 有芒早粳 | Youmangzaogeng | Japonica | 1 | -/- |
| 18 |  | 10-00463 | 铁杆乌 | Tieganwu | Japonica | 1 | -/- |
| 34 |  | 13-01301 | 一支香 | Yizhixiang | Indica | 1 | -/- |
| 52 |  | 17-00435 | 洞庭晚籼 | Dongtingwanxian | Indica | 1 | -/- |
| 57 |  | 18-01067 | 白壳旱禾 | Baikehanhe | Indica | 1 | -/- |
| 66 |  | 20-01734 | 南天纲酒 | Nantiangangjiu | Japonica | 1 | -/- |
| 68 |  | 20-02821 | 中农4号 | Zhongnong 4 | Indica | 1 | -/- |
| 75 |  | 21-00529 | 三七十 | Sanqishi | Japonica | 1 | -/- |
| 88 |  | 21-02089 | 拉木加 | Lamujia | Japonica | 1 | -/- |
| 91 |  | 21-02235 | 鱼眼糯 | Yuyannuo | Japonica | 1 | -/- |
| 92 |  | 21-02619 | 黄皮糯 | Huangpinuo | Japonica | 1 | -/- |
| 95 |  | 21-02851 | 毫香 | Haoxiang | Indica | 1 | -/- |
| 96 |  | 21-02852 | 毫荒腊 | Haohuangla | Indica | 1 | -/- |
| 101 |  | 21-04413 | 半节芒 | Banjiemang | Japonica | 1 | -/- |
| 104 |  | 21-05048 | 细麻线 | Ximaxian | Indica | 1 | -/- |
| 108 |  | 22-00513 | 麻谷糯 | Magunuo | Japonica | 1 | -/- |
| 112 |  | 22-01843 | 红壳折糯 | Hongkezhenuo | Japonica | 1 | -/- |
| 114 |  | 22-02356 | 飞蛾糯2 | Feienuo 2 | Indica | 1 | -/- |
| 121 |  | 24-00195 | 麻谷子 | Maguzi | Japonica | 1 | -/- |
| 151 |  | A00598 | 88B | 88B | Indica | 1 | -/- |
| 159 |  | R0447 | 湖恢628 | Huhui628 | Japonica | 1 | -/- |
| 162 |  | R00604 | JWR221 | JWR221 | Japonica | 1 | -/- |
| 169 |  | ZD-00587 | 桂花黄 | Guihuahuang | Japonica | 1 | -/- |
| 170 |  | ZD-00592 | 苏粳2号 | Sugeng 2 | Japonica | 1 | -/- |
| 183 |  | ZD-01512 | 南特号 | Nantehao | Indica | 1 | -/- |
| 184 |  | ZD-01559 | 秀水115 | Xiushui 15 | Japonica | 1 | -/- |
| 192 |  | ZD-02495 | 早熟香黑 | Zaoshuxianghei | Indica | 1 | -/- |
| 203 |  | ZD-03867 | 四倍体朝6 | Sibeitichao 6 | Japonica | 1 | -/- |
| 206 |  | WT-3 | 旱稻297 | Handao 297 | Japonica | 1 | -/- |
| 219 |  | WT-16 | Azucena | Azucena | Japonica | 1 | -/- |
| 220 |  | WT-17 | IR64 | IR64 | Indica | 1 | -/- |
| 224 |  | WT-21 | Hwayyoung | Hwayyoung | Japonica | 1 | -/- |
| 225 |  | WT-22 | T67 | T67 | Japonica | 1 | -/- |
| 226 |  | WT-23 | IRAT12 | IRAT12 | Japonica | 1 | -/- |
| 227 |  | WT-24 | Rovolele | Rovolele | Indica | 1 | -/- |
|  |  |  |  |  |  |  |  |
| 228 |  |  | Kitaake | Kitaake | Japonica | 1 | -/- |
| 229 |  |  | 湘早籼31 | Xiangzaoxian 31 | Indica | 1 | -/- |
| 230 |  |  | 黄花占 | Huanghuazhan | Indica | 1 | -/- |

- “-/18” the number indicated the data of tiller number investigated in 2011 and 2013 respectively, and “-”indicated the corresponding rice tiller number is not investigated in that year.
