# Supplementary material for: The Copy Number Variation of OsMTD1 Regulates Rice Plant Architecture
Source: Front Plant Sci. 2021 Feb 11;11:620282. doi: 10.3389/fpls.2020.620282 (PMC7905320; doi:10.3389/fpls.2020.620282)
Supplement: Supplementary Table 2 — DNA polymorphism analysis of OsMTD1-located CNV region in various rice cultivars. Nipponbare rep1, Nipponbare rep2: the first and the second sequence of the two DNA segments in Nipponbare genome; ZH11 rep1, ZH11 rep2: the first and the second sequence of the two DNA segments in ZH11 genome; Shuhui 498, 93–11, Minghui 63, RP Bio-226, and Zhenshan 97: the DNA sequence of the OsMTD1-located CNV in corresponding indica cultivar’s genome. [file Table_2.DOCX]

**Supplementary** **Table 2** DNA polymorphism analysis of *OsMTD1*-located CNV region in various rice cultivars

| Rice Cultivar | size (bp) | # SNPs | # deletions | # insertions | % identity |
| --- | --- | --- | --- | --- | --- |
| The sequence of *OsMTD1* located region from Nipponbare rep1 as a reference | | | | | |
| Nipponbare rep1 | 13002 |  |  |  |  |
| Nipponbare rep2 | 13002 | 0 | 0 | 0 | 100 |
| Zhonghua11 rep1 | 12977 | 7 | 23 | 1 | 99.74 |
| Zhonghua11 rep2 | 12992 | 0 | 10 | 0 | 99.92 |
| Shuhui 498 | 13113 | 107 | 10 | 11 | 97.70 |
| The sequence of *OsMTD1* located region from Shuhui 498 as a reference | | | | | |
| Shuhui 498 | 13113 |  |  |  |  |
| 93-11 | 13103 | 0 | 0 | 1 | 99.92 |
| Minghui 63 | 13112 | 0 | 1 | 0 | 99.99 |
| RP Bio-226 | 13103 | 1 | 1 | 0 | 99.92 |
| Zhenshan 97 | 13111 | 0 | 2 | 0 | 99.98 |

**Note:** The polymorphism analysis was conducted in DNAMAN8, and the analysis involved sequences including: Nipponbare rep1: the first sequence of the two DNA segments in Nipponbare genome; Nipponbare rep2: the second sequence of the two DNA segments in Nipponbare genome; ZH11 rep1: the first sequence of the two DNA segments in ZH11 genome; ZH11 rep2: the second sequence of the two DNA segments in ZH11 genome; Shuhui 498, 93-11, Minghui 63, RP Bio-226, Zhenshan 97: the corresponding DNA sequence of the *OsMTD1*-located CNV in corresponding *indica* cultivar’s genome.
