# Supplementary material for: The Copy Number Variation of OsMTD1 Regulates Rice Plant Architecture
Source: Front Plant Sci. 2021 Feb 11;11:620282. doi: 10.3389/fpls.2020.620282 (PMC7905320; doi:10.3389/fpls.2020.620282)
Supplement: Supplementary Table 3 — The result of Distance and Homology matrix analysis. Evolutionary analysis were conducted in DNAMAN8, and the analysis involved eight sequences: Nipponbare rep1: the first sequence of the two DNA segments in Nipponbare genome; ZH11-rep1, ZH11-rep2: the first and the second sequence of the two DNA segments in ZH11 genome; Shuhui 498, 93–11, Minghui 63, RP Bio-226, and Zhenshan 97: the DNA sequence of the OsMTD1-located CNV in corresponding indica cultivar’s genome. [file Table_3.DOCX]

**Supplementary** **Table 3** The result of Distance & Homology matrix analysis

**Distance matrix of 8 sequences**

Nipponbare-rep 0

ZH11-rep1 0.001 0

ZH11-rep2 0.000 0.001 0

93-11-rep 0.009 0.010 0.009 0

Minghui63-rep 0.009 0.010 0.009 0.000 0

RPBio226-rep 0.009 0.010 0.009 0.000 0.000 0

Shuhui498-rep 0.009 0.010 0.009 0.000 0.000 0.000 0

Zhenshan97-rep 0.009 0.010 0.009 0.000 0.000 0.000 0.000 0

**Homology matrix of 8 sequences**

Nipponbare-rep 100%

ZH11-rep1 99.9% 100%

ZH11-rep2 100.0% 99.9% 100%

93-11-rep 99.1% 99.0% 99.1% 100%

Minghui63-rep 99.1% 99.0% 99.1% 100.0% 100%

RPBio226-rep 99.1% 99.0% 99.1% 100.0% 100.0% 100%

Shuhui498-rep 99.1% 99.0% 99.1% 100.0% 100.0% 100.0% 100%

Zhenshan97-rep 99.1% 99.0% 99.1% 100.0% 100.0% 100.0% 100.0% 100%

**Note:** The number of base differences per site from between sequence are shown. Evolutionary analyses were conducted in DNAMAN8, and the analysis involved 8 sequences: Nipponbare rep1: the first sequence of the two DNA segments in Nipponbare genome; ZH11-rep1: the first sequence of the two DNA segments in ZH11 genome; ZH11-rep2: the second sequence of the two DNA segments in ZH11 genome; Shuhui498-rep: the corresponding DNA sequence of the *OsMTD1* located CNV in *indica* cv. Shuhui 498 genome; 93-11-rep: the corresponding DNA sequence of the *OsMTD1* located CNV in *indica* cv. 93-11 genome; Minghui 63: the corresponding DNA sequence of the *OsMTD1* located CNV in *indica* cv. Minghui 63 genome; RP Bio-226: the corresponding DNA sequence of the *OsMTD1* located CNV in *indica* cv. RP Bio-226 genome; Zhanshan97-rep: the corresponding DNA sequence of the *OsMTD1* located CNV in *indica* cv. Zhenshan 97 genome.
