# Supplementary material for: The Copy Number Variation of OsMTD1 Regulates Rice Plant Architecture
Source: Front Plant Sci. 2021 Feb 11;11:620282. doi: 10.3389/fpls.2020.620282 (PMC7905320; doi:10.3389/fpls.2020.620282)
Supplement: Supplementary Table 4 — The mutation information of OsMTD1 in different CRISPR/Cas9 editing lines. [file Table_4.DOCX]

**Supplementary** **Table 4** The mutation information of *OsMTD1* in different CRISPR/Cas9 editing lines

| **Line Number** | **Mutation information** | **Consequence** |
| --- | --- | --- |
| A-3 | Inserted C | A single-base insertion results in a frame-shift mutation |
| A-8 | Inserted T | A single-base insertion results in a frame-shift mutation |
| A-16 | Deleted G | A single-base deletion results in a frame-shift mutation |
| A-27 | Deleted T | A single-base deletion results in a frame-shift mutation |
| A-42 | Inserted A | A single-base insertion results in a frame-shift mutation |
| A-44 | Inserted G | A single-base insertion results in a frame-shift mutation |
