# Supplementary material for: The Copy Number Variation of OsMTD1 Regulates Rice Plant Architecture
Source: Front Plant Sci. 2021 Feb 11;11:620282. doi: 10.3389/fpls.2020.620282 (PMC7905320; doi:10.3389/fpls.2020.620282)
Supplement: Supplementary Table 5 — The OsMTD1 level in different overexpression lines. [file Table_5.DOCX]

**Supplementary** **Table 5** The *OsMTD1* expression level in different overexpression lines

| **Line Number** | **Increased times compared with wildtype** |
| --- | --- |
| OE-15 | 16188 |
| OE-16 | 22178 |
| OE-19 | 14500 |
| OE-20 | 3.1 |
| OE-26 | 3.4 |
| OE-27 | 22 |
